# Spike-triggered covariance: geometric proof, symmetry properties, and extension beyond Gaussian stimuli

**DOI:** 10.1007/s10827-012-0411-y

**Published:** 2012-07-15

**Authors:** Inés Samengo, Tim Gollisch

**Affiliations:** 1 Centro Atómico Bariloche and Instituto Balseiro, (8400) San Carlos de Bariloche, Río Negro, Argentina; 2 Department of Ophthalmology and Bernstein Center for Computational Neuroscience Göttingen, Georg-August University Göttingen, 37073 Göttingen, Germany

**Keywords:** Covariance analysis, Spike-triggered average, Receptive field, Linear-nonlinear model

## Abstract

The space of sensory stimuli is complex and high-dimensional. Yet, single neurons in sensory systems are typically affected by only a small subset of the vast space of all possible stimuli. A proper understanding of the input–output transformation represented by a given cell therefore requires the identification of the subset of stimuli that are relevant in shaping the neuronal response. As an extension to the commonly-used spike-triggered average, the analysis of the spike-triggered covariance matrix provides a systematic methodology to detect relevant stimuli. As originally designed, the consistency of this method is guaranteed only if stimuli are drawn from a Gaussian distribution. Here we present a geometric proof of consistency, which provides insight into the foundations of the method, in particular, into the crucial role played by the geometry of stimulus space and symmetries in the stimulus–response relation. This approach leads to a natural extension of the applicability of the spike-triggered covariance technique to arbitrary spherical or elliptic stimulus distributions. The extension only requires a subtle modification of the original prescription. Furthermore, we present a new resampling method for assessing statistical significance of identified relevant stimuli, applicable to spherical and elliptic stimulus distributions. Finally, we exemplify the modified method and compare it to other prescriptions given in the literature.

## Introduction

Neurons in sensory systems are often exquisitely tuned to specific stimulus features. Thus, a first step in the characterization of their input–output transformation is to identify which aspects of the stimulus affect a neuron’s activity level and which do not. As the space of possible stimuli is typically high-dimensional, an exhaustive exploration of all candidate stimuli appears impractical. But fortunately for neuroscientists, individual neurons often seem to be remarkably selective and only care about subspaces of low dimensionality. The identification of such low-dimensional spaces of relevant stimuli is a crucial challenge in sensory neuroscience.

In the simplest scenario, an analysis may aim at identifying a single relevant dimension in stimulus space, corresponding to a particular stimulus feature. This is suited, for example, for neurons whose response properties are well captured by their receptive fields. Neurons in the early visual system are often described by their spatio-temporal receptive fields (Hartline 1940; Kuffler 1953; Hubel and Wiesel 1962; Meister and Berry 1999; Reich et al. 2000) and neurons in the auditory system by their spectro-temporal receptive fields (Eggermont et al. 1983a, b; Kim and Young 1994; deCharms et al. 1998). A standard technique for assessing the receptive field from electrophysiological experiments is to measure the spike-triggered average (STA) under stimulation with a broad-band signal (de Boer and Kuyper 1968; Bryant and Segundo 1976; de Boer and de Jongh 1978; Eggermont et al. 1983a; de Ruyter van Steveninck and Bialek 1988; Chichilnisky 2001; Nykamp and Ringach 2002; Schwartz et al. 2006), typically white noise. The technique largely owes its immense popularity to its computational simplicity, obviating the need for complex parameter fitting. The analysis consists of collecting all stimulus segments that precede a spike and averaging them together. This amounts to correlating the measured spikes with the applied stimulus, so the method also falls under the name of “reverse correlation”.

In many cases, however, a single stimulus feature is insufficient to describe a neuron’s response characteristics. If the cell is sensitive to several features and pools them in a nonlinear fashion, its stimulus–response relation may not be well captured by just the receptive field. A well-known example is the energy model of complex cells in visual cortex (Adelson and Bergen 1985), which comprises two spatial Gabor filters whose outputs are squared before summation. As the model responds equally to positive and negative visual contrasts, the STA is identical to zero. Other methods are thus required to characterize neurons with symmetric response characteristics. In other cases, the STA may provide an approximate model of the cell’s stimulus–response relation, but adding further stimulus components considerably improves the accuracy of the model.

For these reasons, spike-triggered covariance (STC) analysis has emerged as a popular extension of the STA (Bryant and Segundo 1976; de Ruyter van Steveninck and Bialek 1988; Paninski 2003; Bialek and de Ruyter van Steveninck 2005; Brenner et al. 2000; Schwartz et al. 2002; Rust et al. 2004; Simoncelli et al. 2004). In STC analysis, the stimulus segments that precede a spike are characterized through a principal component analysis, which allows the extraction of multiple relevant stimulus dimensions. The basic idea is to detect differences in variance between the distribution of spike-producing stimulus segments and the prior distribution of all stimulus segments. Although alternative techniques exist that are applicable under more general stimulation (Paninski 2003; Paninski et al. 2004; Sharpee et al. 2004; Pillow and Simoncelli 2006; Park and Pillow 2011) or in connection with additional post-spike dynamics (Keat et al. 2001; Aldworth et al. 2005; Pillow et al. 2005, 2008; Dimitrov and Gedeon 2006; Gollisch 2006), STC analysis has retained considerable popularity, just like the STA, because of its relative computational simplicity.

The statistics of the applied stimulus play an important role for applying STA and STC analysis. For neurons whose firing probability depends on a single stimulus direction, the STA provides a consistent and unbiased estimator of the relevant direction when the probability distribution of all applied stimuli displays spherical symmetry (Chichilnisky 2001; Paninski 2003). This condition states that all stimulus segments that have the same magnitude (i.e. the same Euclidean norm) must also have the same probability of occurrence. If the distribution of stimuli is not spherically symmetric, the STA is typically biased, as it deviates in a systematic fashion from the relevant stimulus dimension. Moreover, as this bias does not depend on the amount of available data, the estimate provided by the STA is then not consistent. The simplest way to fulfill the criterion of spherical symmetry is to draw each stimulus component from the same Gaussian distribution. But there are, of course, many other ways to construct spherical distributions of stimuli.

Via a simple extension, it is straightforward to apply STA analysis also to stimuli with an elliptic distribution. Here, *elliptic distribution* refers to any distribution that can be obtained from a spherical distribution by a linear transformation that stretches or compresses individual directions in stimulus space. The spectrum of the stimulus is therefore non-white, and different stimulus components are correlated with each other. To apply STA analysis, the stretching transformation simply has to be “undone” after the spike-producing stimulus segments have been averaged (Theunissen et al. 2001; Paninski 2003; Schwartz et al. 2006).

Surprisingly, the requirements concerning the stimulus distribution are more restrictive for STC analysis, where stimuli need to follow not just a spherically symmetric, but a Gaussian distribution to guarantee that the analysis provides a consistent estimator of the relevant stimulus space (Paninski 2003; Sharpee et al. 2004; Simoncelli et al. 2004; Schwartz et al. 2006). Given the otherwise tight analogy between STA and STC analysis, this difference appears puzzling. For STA analysis, the requirement of a spherically symmetric stimulus distribution is best understood in a geometric picture of why the technique works (Chichilnisky 2001). The insight and intuition supplied by the geometric proof thus calls for a similar perspective on STC analysis.

Here, we provide such a geometric derivation for STC analysis, leading to a simple proof of why the technique works, that is, of the consistency of the method. Furthermore, the geometric approach highlights the importance of spherical symmetry also for STC analysis and suggests a simple modification of the procedure that makes it applicable to stimulus ensembles with general spherical symmetry, not necessarily Gaussian. We further extend this approach to arbitrary elliptic stimulus distributions, containing correlations between different stimulus components. To facilitate identification of relevant stimulus dimensions for finite data sets, we then introduce a new statistical test for significance of relevant stimulus dimensions. Finally, we compare the obtained prescription with others that have been used in the literature.

## Linear-nonlinear models

As for many other analyses of neuronal stimulus–response relationships, describing sensory stimuli as vectors in a (potentially high-dimensional) space of stimuli has provided a useful perspective for spike-triggered average and spike-triggered covariance analyses. We here denote a stimulus as a column vector $\boldsymbol {s}$ in an *N*-dimensional space,

1 $$ \boldsymbol {s} = \left( \begin{array}{c} s_1\\ s_2\\ \vdots \\ s_N \end{array} \right). $$

**Fig. 1:**
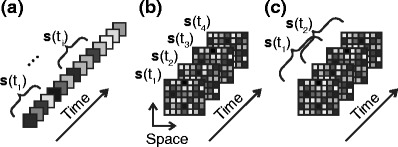
Vectorial representation of stimuli. Different components represent the value of a stimulus at different time points (**a**), different spatial locations (**b**), or both (**c**). Spatial binnings (**b**) can also be used to represent any other non-temporal aspect of the stimulus. Spatial and temporal dimensions may be combined into a unified spatio-temporal representation (**c**), for example, to study visual spatio-temporal receptive fields

The individual components of $\boldsymbol {s}$ can represent, for example, the strength of stimulation at different points in time (Fig. 1(a)), different spatial locations (Fig. 1(b)), or a combination of the two (Fig. 1(c)). Of course, space could also be supplemented or substituted by any other relevant stimulus attribute, for example, spectral components. Pure temporal binnings (Fig. 1(a)) represent the simplest scenario, when only the history of an otherwise one-dimensional stimulus needs to be taken into account. They are used, for example, when neurons in the visual system are stimulated with changing ambient light intensity that contains no spatial structure, or when an auditory neuron is analyzed for its responses to the temporal modulation (i.e. the envelope) of a pure tone. The stimulus vector $\boldsymbol {s}$ is then defined at discrete times *t*, and the components of $\boldsymbol {s}(t)$ represent the stimulus strength within time bins of length Δ*t* during the recent past, for example, by sampling time discretely,

2 $$ s_n(t) = s(t-(N-n)\cdot \Delta t),\; {\rm for }\; n=1,\ldots,N. $$

The dimension *N* of the vectors should be chosen large enough to encompass the relevant stimulus structure.

The methods of spike-triggered average and spike-triggered covariance constitute rigorous estimators of neuronal filtering characteristics when the spike-generating process is well described by a linear–nonlinear (LN) model. In such models, the stimulus $\boldsymbol {s}$ is first filtered by one or several linear filters $\boldsymbol {k}_m$. We denote the number of filters by *M*. Typically, there are far fewer filters than stimulus dimensions, *M* ≪ *N*. The filters are, just as the stimuli, represented by *N*-dimensional vectors

3 $$ \boldsymbol {k}_m = \left( \begin{array}{c} k_{m,1}\\ k_{m,2}\\ \vdots \\ k_{m,N} \end{array} \right). $$

Applying the filter $\boldsymbol {k}_m$ to a stimulus $\boldsymbol {s}$ yields

4 $$ \boldsymbol {k}_m^T \ \boldsymbol {s} = \sum\limits_{i=1}^N k_{m,i} \cdot s_i. $$

The filtered signals are then transformed into the probability of generating a spike in response to stimulus *s* , $P({\rm spike}|\boldsymbol {s})$, where the variable “spike” here takes the value 1 for a generated spike or 0 for no spike. The transformation occurs through a static nonlinear function *φ* with *M* input variables,

5 $$ P({\rm spike} | \boldsymbol {s}) = \varphi \left(\boldsymbol {k}_1^T \boldsymbol {s},\ \boldsymbol {k}_2^T \boldsymbol {s},\ \dots,\ \boldsymbol {k}_M^T \boldsymbol {s} \right). $$

According to Eq. (5), the stimulus affects the spike probability only through its projections onto the filters $\boldsymbol {k}_m$. The filters therefore demarcate relevant directions in stimulus space (Paninski 2003), corresponding to stimulus features that affect the spike probability. The subspace spanned by these filters, ${\cal K}=span(\boldsymbol {k}_1,\ \boldsymbol {k}_2,\ \ldots,\ \boldsymbol {k}_M)$, is called the *relevant subspace*. Any stimulus vector that is orthogonal to the relevant subspace does not affect the spiking probability because it does not affect the inputs into the function *ϕ*. The orthogonal complement of ${\cal K}$ therefore constitutes the *irrelevant subspace* ${\cal K}_\perp$. The aim of spike-triggered covariance analysis is to identify the relevant and the irrelevant stimulus subspaces.

The stimuli $\boldsymbol {s}$ that enter the LN model come from a prior stimulus distribution $P(\boldsymbol {s})$, typically determined by the experimenter when presenting sensory stimuli. Both STA and STC analysis rely on comparing this prior stimulus distribution to the distribution of stimuli that precede spikes, $P(\boldsymbol {s}|{\rm spike})$.

Often, the prior stimulus distribution is chosen to be Gaussian white noise with fixed variance *σ* ^2^,

6 $$ P(\boldsymbol {s}) = \frac{1}{\left(2 \pi \sigma^2 \right)^{N/2}} \ \exp \left(-\frac{1}{2\sigma^2} \ \boldsymbol {s}^T \boldsymbol {s}\right). $$

The Gaussian white noise distribution has the remarkable property that, in addition to being spherically symmetric, it may be written as a product of distributions, one for each stimulus component,

7 $$ P(\boldsymbol {s}) = \prod\limits_{i=1}^N \frac{1}{\sqrt{2 \pi \sigma^2 }} \ {\rm exp}\left(-\frac{{s_i}^2}{2 \sigma^2} \right). $$

Thus, each stimulus component is independent of the others. If the spiking probability only depends on a few stimulus directions, the stimulus distributions $P(\boldsymbol {s})$ and $P(\boldsymbol {s}|{\rm spike})$ only differ along these directions. Along any orthogonal stimulus direction, the two distributions coincide. The invariance along irrelevant directions forms the basis of spike-triggered analysis for Gaussian white noise stimuli: Relevant stimulus directions are identified as those where the distribution of spike-generating stimuli differs from the prior distribution.

The Gaussian white stimulus constitutes a special case of a distribution with spherical symmetry, for which the prior distribution $P(\boldsymbol {s})$ depends only on the absolute value $|\boldsymbol {s}|=\sqrt{\boldsymbol {s}^T \boldsymbol {s}}$ of its argument, that is,

8 $$ P(\boldsymbol {s}) = P(|\boldsymbol {s}|). $$

**Fig. 2:**
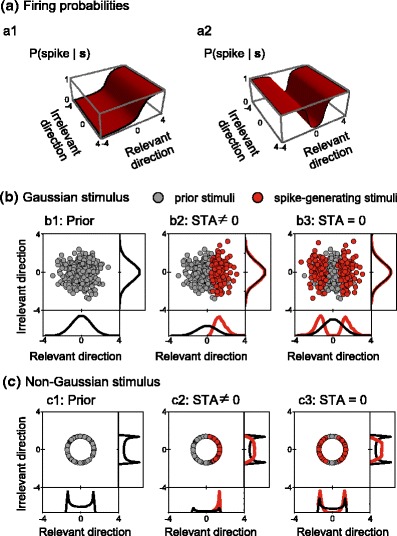
Two-dimensional examples of a spike-generating process. (**a**) Non-linear functions *ϕ* used to generate spikes in the examples below. (**b**) Spherical Gaussian prior stimulus distribution. (**c**) Spherical non-Gaussian prior stimulus distribution. In all cases, the probability to generate spikes only depends on the relevant direction (*horizontal axis*). (**b1**) and (**c1**) Ensemble of prior stimulus vectors. (**b2**) and (**c2**) Prior (*gray dots*) and spike-generating (*red dots*) stimuli obtained from the nonlinearity in panel (**a1**), resulting in a spike-triggered average that is different from zero. (**b3**) and (**c3**) Prior (*gray dots*) and spike-generating (*red dots*) stimuli obtained from the nonlinearity in panel (**a2**), resulting in STA = 0. The stimulus distributions along each dimension are shown in the side panels. If the distribution is Gaussian, $P(\boldsymbol {s}) = P(\boldsymbol {s} | {\rm spike})$ along the irrelevant direction. For non-Gaussian stimuli, $P(\boldsymbol {s}) \ne P(\boldsymbol {s} | {\rm spike})$ along both relevant and irrelevant directions

For non-Gaussian stimuli, different stimulus directions are not independent of one another. As a consequence, the distributions $P(\boldsymbol {s})$ and $P(\boldsymbol {s}|{\rm spike})$ not only differ inside the relevant space ${\cal K}$, but typically also along the irrelevant directions in ${\cal K}_\perp$. In Fig. 2, the prior stimulus distribution and the spike-triggered stimulus distribution are shown for 2-dimensional toy examples. In each case, the spike probability only depends on one of the two stimulus components, as illustrated by the nonlinear functions *φ* in Fig. 2(a). The relevant and the irrelevant direction in stimulus space are defined by the contour curves of *φ*: Along the relevant direction, *φ* varies, whereas it always remains constant along the orthogonal irrelevant direction. If stimuli are drawn from a Gaussian distribution, the prior and spike-triggered distributions are identical along the irrelevant direction (Fig. 2(b)). When stimuli come from a spherically symmetric, non-Gaussian, annular distribution, however, the two distributions differ also along the irrelevant direction (Fig. 2(c)). The annular shape of the prior distribution imposes a constraint, linking the values of relevant and irrelevant components. A change in variance along the relevant direction hence induces a change in variance along the irrelevant direction as well. Consequently, at first sight, it may seem that STC analysis would be inapplicable to these cases. Simply looking for directions in stimulus space along which the variance of $P(\boldsymbol {s}|{\rm spike})$ differs from the variance of $P(\boldsymbol {s})$ would lead to the erroneous classification of the irrelevant direction as relevant. Below we show, however, that the more realistic case of higher-dimensional stimuli brings in additional structure not apparent in this 2-dimensional toy example. The clue lies in the fact that in *all* irrelevant directions, the variances of $P(\boldsymbol {s})$ and $P(\boldsymbol {s}|{\rm spike})$ differ by exactly the same amount. This constancy typically makes the irrelevant directions distinguishable from the relevant ones, even in the non-Gaussian case.

## Geometric picture of STC analysis for spherically symmetric stimulus distributions

### Basic definitions

We first consider spike-triggered covariance analysis for stimuli that have a spherically symmetric prior distribution, Gaussian or not. Extensions beyond the spherical case are discussed in Section 4. To simplify the notation, we assume that the mean value of the prior stimulus distribution has already been subtracted from all stimulus vectors, that is, we choose the origin of the coordinate system so that the prior distribution of stimuli $P(\boldsymbol {s})$ has zero mean,

9 $$ \int {\rm d}\boldsymbol {s} \ P(\boldsymbol {s}) \ \boldsymbol {s} = \boldsymbol {0}. $$

The prior covariance matrix *C*_*p*_ of a spherically symmetric stimulus distribution is proportional to the unit matrix. Here, we set the units in stimulus space such that *C*_*p*_ coincides with the identity matrix,

10 $$ C_p = \int {\rm d}\boldsymbol {s} \ P(\boldsymbol {s}) \ \boldsymbol {s} \ \boldsymbol {s}^T = \mathbb{I}_{N \times N}, $$

where the product $\boldsymbol {s}\ \boldsymbol {s}^T$ is the matrix

11 $$ \boldsymbol {s}\ \boldsymbol {s}^T = \left( \begin{array}{cccc} s_1 s_1 & s_1 s_2 & \cdots & s_1 s_N \\ s_1 s_2 & s_2 s_2 & \cdots & s_2 s_N \\ \vdots & \vdots & \ddots & \vdots \\ s_1 s_N & s_2 s_N & \cdots & s_N s_N \end{array} \right). $$

A neuron with a firing probability given by Eq. (5) is only sensitive to the projection of the actual stimulus $\boldsymbol {s}$ on the relevant space ${\cal K}$. Covariance analysis provides a systematic procedure to find ${\cal K}$, based on the first two moments of the distribution $P(\boldsymbol {s} | {\rm spike})$. The spike-triggered average $\langle \boldsymbol {s} \rangle$ is the first moment of the distribution of spike-triggered stimuli

12 $$ \begin{array}{rll} \langle \boldsymbol {s} \rangle &=& \int {\rm d}\boldsymbol {s} \ P(\boldsymbol {s} | {\rm spike}) \ \boldsymbol {s} \\ &=& \frac{1}{r} \int {\rm d}\boldsymbol {s} \ P({\rm spike} | \boldsymbol {s}) \ P(\boldsymbol {s}) \ \boldsymbol {s} , \end{array} $$

where *r* is the total average spike probability per stimulus presentation,

13 $$ r = \int {\rm d}\boldsymbol {s} \ P({\rm spike} | \boldsymbol {s}) \ P(\boldsymbol {s}), $$

and the second equality in Eq. (12) derives from Bayes’ rule. This rearrangement makes the dependence on the prior stimulus distribution *P*(**s)** explicit, which will turn out useful in the derivations below. Throughout this paper, all averages $\langle \cdot \rangle$ are calculated over the distribution of spike-triggered stimuli, $P(\boldsymbol {s} | {\rm spike})$.

When working with experimental data, the distribution $P(\boldsymbol {s}|{\rm spike})$ is not directly available. Therefore, one typically works with the sample STA $\hat{\langle \boldsymbol {s}\rangle}$, which is the average of all stimulus segments $\boldsymbol {s}(t_{\rm spike})$ that precede the measured spikes at times *t*_spike_,

14 $$ \hat{\langle\boldsymbol {s}\rangle} = \frac{1}{N_{\rm spikes}}\sum_{t_{\rm spike}}\boldsymbol {s}\left(t_{\rm spike}\right) . $$

For large enough data sets, the law of large numbers ensures that the stimulus segments $\boldsymbol {s}(t_{\rm spike})$ sample the spike-triggered distribution $P(\boldsymbol {s}|{\rm spike})$ thoroughly, so that $\hat{\langle\boldsymbol {s}\rangle}$ approaches $\langle\boldsymbol {s}\rangle$, as defined in Eq. (12).

The covariance of the distribution of spike-triggered stimuli, $P(\boldsymbol {s} | {\rm spike})$, is captured by the spike-triggered covariance matrix

15 $$ \begin{array}{rll} C_s &=& \left\langle \left( \boldsymbol {s} - \langle \boldsymbol {s} \rangle \right) \left( \boldsymbol {s} - \langle \boldsymbol {s} \rangle \right)^T \right\rangle = \left\langle \boldsymbol {s}\boldsymbol {s}^T \right\rangle - \langle\boldsymbol {s}\rangle \langle\boldsymbol {s} \rangle^T \\ &=& \frac{1}{r} \int {\rm d}\boldsymbol {s} \ P({\rm spike} | \boldsymbol {s}) \ P(\boldsymbol {s}) \ \boldsymbol {s} \ \boldsymbol {s}^T \ - \ \langle \boldsymbol {s} \rangle \langle \boldsymbol {s} \rangle^T . \end{array} $$

This matrix is typically estimated from experimental data by the sample covariance matrix

16 $$ \begin{array}{rll} \hat{C_s} &=& \frac{1}{N_{\rm spikes}-1} \\ && \times \sum\limits_{t_{\rm spike}} \left(\boldsymbol {s}\left(t_{\rm spike}\right)-\hat{\langle\boldsymbol {s}\rangle}\right) \left(\boldsymbol {s}\left(t_{\rm spike}\right)-\hat{\langle\boldsymbol {s}\rangle}\right)^T . \end{array} $$

Again, for large enough data sets, $\boldsymbol {s}(t_{\rm spike})$ samples the distribution $P(\boldsymbol {s}|{\rm spike})$ thoroughly, so that $\hat{C_s}$ approaches *C*_*s*_ as defined in Eq. (15).

Standard STC analysis is based on the fact that when the prior stimuli are drawn from a Gaussian white distribution and sufficient amounts of data are available, the diagonalization of *C*_*s*_ yields two types of eigenvalues. Those corresponding to irrelevant directions are equal to 1, that is, to the variance of the prior stimulus distribution. The ones corresponding to relevant directions may have any (non-negative) value, depending on the variance along each direction.

Limited sampling adds noise to the eigenvalues so that those corresponding to irrelevant dimensions do not all lie exactly at unity, but scatter around this level. Statistical methods can then be used to assess whether deviations from unity significantly indicate the existence of a relevant direction (Touryan et al. 2002; Rust et al. 2005; Schwartz et al. 2006). A relevant stimulus direction with an eigenvalue that happens to lie very close to unity, however, may be missed by the method.

Even in the limit of infinite amounts of data, however, relevant directions could escape detection by the eigenvalue analysis of the STC matrix. A deviation from unity is a sufficient, but not a necessary condition for an eigenvalue to denote a relevant direction (Paninski 2003; Pillow and Simoncelli 2006); its eigenvalue may happen to lie exactly at unity. This can occur, for example, when the prior stimulus distribution is Gaussian and the nonlinearity *φ* is an exponential function of one of its arguments because exponential nonlinearities leave the variance along the corresponding relevant direction unchanged. This limitation of STC analysis reflects the fact that the method is based entirely on second-order statistics of spike-triggered stimuli. Typically, a simple remedy is thus to explicitly include the STA in the identification of the relevant stimulus space (Rust et al. 2004, 2005; Simoncelli et al. 2004; Schwartz et al. 2006), as relevant directions that do not show a difference in stimulus variance between prior and spike-triggered stimulus ensemble can be expected to show a difference in the stimulus average.

These remarks also apply to STC analysis for non-Gaussian, spherically symmetric stimulus distributions, as discussed below. Therefore, the possibility that a relevant stimulus direction might not be revealed through the spectrum of eigenvalues must be kept in mind. Having said this, for simplicity we assume in the following that eigenvalues of relevant directions do not “by chance” coincide with the eigenvalues of irrelevant directions, as again, this can generally be picked up by analyzing the STA. In addition, the issue of limited sampling and significance testing will be put off until Section 5.

### A geometric derivation

As a basis for applying spike-triggered covariance analysis to any stimulus distribution with spherical symmetry, including non-Gaussian stimuli, we show that the irrelevant space is spanned by eigenvectors of *C*_*s*_ with the same degenerate eigenvalue. It then follows that the relevant space can be identified as the subspace that is spanned by eigenvectors of *C*_*s*_ whose eigenvalues deviate from the baseline level of (typically a large number of) degenerate eigenvalues. In this derivation, we work directly with the probability distribution of spike-triggered stimuli and thus do not consider noise from finite sampling. We thereby provide a proof of consistency of the method, which means that it yields the correct relevant subspace in the limit of infinite data sampling.

The key point of the proof is to show that any vector of the irrelevant space is an eigenvector of *C*_*s*_. This statement is geometrically derived below and immediately implies that the whole irrelevant subspace ${\cal K}_\perp$ is a degenerate eigenspace of *C*_*s*_, so that all stimulus vectors of the irrelevant space have the same eigenvalue: Consider two non-parallel vectors $\boldsymbol {v}_1$ and $\boldsymbol {v}_2$ that belong to the irrelevant space. According to the statement above, they must be eigenvectors of *C*_*s*_ with eigenvalues *α*_1_ and *α*_2_. Their sum lies also within the irrelevant space and is thus also an eigenvector. Let *β* be the eigenvalue of $\boldsymbol {v}_1 + \boldsymbol {v}_2$. Now, the identity $C_s \cdot (\boldsymbol {v}_1 + \boldsymbol {v}_2) = \alpha_1 \boldsymbol {v}_1 + \alpha_2 \boldsymbol {v}_2 = \beta (\boldsymbol {v}_1 + \boldsymbol {v}_2 )$ can only be fulfilled for *α*_1_ = *α*_2_ = *β*.

To prove that each (non-zero) vector of the irrelevant space is an eigenvector of *C*_*s*_, we draw out an argument analogous to the geometric proof of consistency of STA analysis by Chichilnisky (2001). Specifically, we want to show that for any $\boldsymbol {v} \in {\cal K}_\perp$

17 $$ \begin{array}{rll} C_s \boldsymbol {v} &=& \frac{1}{r} \int {\rm d}\boldsymbol {s} \ P({\rm spike} | \boldsymbol {s}) \ P(\boldsymbol {s}) \ \boldsymbol {s} \ \boldsymbol {s}^T \boldsymbol {v} - \langle\boldsymbol {s}\rangle \langle\boldsymbol {s} \rangle^T \boldsymbol {v} \\ &=& \lambda \boldsymbol {v} \end{array} $$

with a real eigenvalue *λ*. As a first step, we show that the spike-triggered average $\langle \boldsymbol {s} \rangle$ belongs to ${\cal K}$. It follows that $\langle \boldsymbol {s} \rangle$ is perpendicular to $\boldsymbol {v}$, so that the $\langle \boldsymbol {s} \rangle \langle \boldsymbol {s} \rangle^T \boldsymbol {v}$-term in Eq. (17) yields zero because $\langle \boldsymbol {s} \rangle^T \boldsymbol {v} = 0$.

**Fig. 3:**
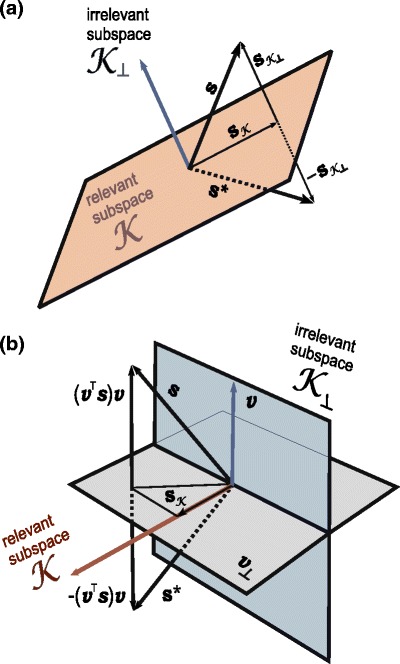
Geometry of the vectors involved in the consistency proof. (**a**): Each vector $\boldsymbol {s}$ has a mirror image $\boldsymbol {s}^*$ with respect to the relevant space ${\cal K}$. When integrating Eq. (12), the components $\boldsymbol {s}_{{\cal K}_\perp}$ and $-\boldsymbol {s}_{{\cal K}_\perp}$ cancel out, so $\langle \boldsymbol {s} \rangle \in {\cal K}$. (**b**): *v* is an arbitrary (normalized) vector in ${\cal K}_\perp$; $\boldsymbol {v}_\perp$ is its orthogonal complement. Each vector $\boldsymbol {s}$ has a mirror image $\boldsymbol {s}^*$ with respect to $\boldsymbol {v}_\perp$. The projection of $\boldsymbol {s}$ onto the relevant subspace ${\cal K}$ is $\boldsymbol {s}_{\cal K}$ and coincides with the projection of $\boldsymbol {s}^*$ onto ${\cal K}$. The difference $\boldsymbol {s} - \boldsymbol {s}^*=2(\boldsymbol {v}^T \boldsymbol {s})\boldsymbol {v}$ is proportional to $\boldsymbol {v}$

To this end, we essentially repeat the argument of Chichilnisky (2001) and thus summarize the derivation here only briefly: For every stimulus $\boldsymbol {s}$, a unique counterpart $\boldsymbol {s}^*$ can be found by taking the mirror image of $\boldsymbol {s}$ with respect to the relevant subspace ${\cal K}$ (Fig. 3(a)). Concretely, with $\boldsymbol {s}_{\cal K}$ denoting the projection of $\boldsymbol {s}$ onto ${\cal K}$ and $\boldsymbol {s}_{{\cal K}_\perp} = \boldsymbol {s}-\boldsymbol {s}_{\cal K}$ denoting the projection of $\boldsymbol {s}$ onto ${\cal K}_\perp$, we have

18 $$ \boldsymbol {s}^* = \boldsymbol {s} - 2 \boldsymbol {s}_{{\cal K}_\perp}. $$

The vectors $\boldsymbol {s}$ and $\boldsymbol {s}^*$ have equal length, so their probabilities within the stimulus ensemble are the same, $P(\boldsymbol {s})=P(\boldsymbol {s}^*)$. Since their projections on ${\cal K}$ are the same, the associated spike probabilities are also equal, $P({\rm spike}|\boldsymbol {s})=P({\rm spike}|\boldsymbol {s}^*)$. Therefore, in calculating the spike-triggered average, $\boldsymbol {s}$ and $\boldsymbol {s}^*$ are weighted equally in Eq. (12). Given that, by construction, the components of $\boldsymbol {s}$ and $ \boldsymbol {s} ^*$ orthogonal to ${\cal K}$ are equal with opposite sign (Fig. 3(a)), these components cancel out in the STA for all pairs $( \boldsymbol {s} ,  \boldsymbol {s} ^*)$. As a consequence, the spike-triggered average $\langle  \boldsymbol {s}  \rangle$ has no component orthogonal to ${\cal K}$ and thus lies in the relevant subspace.

As the $\langle  \boldsymbol {s} \rangle\langle  \boldsymbol {s} \rangle^T \boldsymbol {v}$-term in Eq. (17) vanishes, we now have to show the eigenvalue property of $\boldsymbol {v}$ for the integral term of the equation. To do so, we use a geometric argument very similar to the one above for the STA. We consider a vector $\boldsymbol {v}$ from the irrelevant subspace ${\cal K}_\perp$ (Fig. 3(b)). Let us denote the hyperplane that is orthogonal to $\boldsymbol {v}$ by $\boldsymbol {v}_\perp$. Now, for every stimulus vector $ \boldsymbol {s} $, a unique vector $ \boldsymbol {s} ^*$ can be found that is the mirror image of $ \boldsymbol {s} $ with respect to the hyperplane $\boldsymbol {v}_\perp$, see Fig. 3(b). Assuming that $\boldsymbol {v}$ has unit length, $ \boldsymbol {s} ^*$ is simply obtained as

19 $$  \boldsymbol {s} ^* =  \boldsymbol {s} \ -\ 2\ (\boldsymbol {v}^T  \boldsymbol {s} )\ \boldsymbol {v}. $$

Again, $ \boldsymbol {s} $ and $ \boldsymbol {s} ^*$ have equal length so that $P( \boldsymbol {s} )=P( \boldsymbol {s} ^*)$. Also, the projections of $ \boldsymbol {s} $ and $ \boldsymbol {s} ^*$ on the relevant subspace ${\cal K}$ are identical because $ \boldsymbol {s} $ and $ \boldsymbol {s} ^*$ have the same projections on $\boldsymbol {v}_\perp$ and because ${\cal K}$ is a subspace of $\boldsymbol {v}_\perp$. Thus, the spike probabilities for these two stimuli are the same: $P({\rm spike}| \boldsymbol {s} ) = P({\rm spike}| \boldsymbol {s} ^*)$. We can therefore perform the integral in Eq. (17) over $ \boldsymbol {s} ^*$ instead of over $ \boldsymbol {s} $, or alternatively, substitute $ \boldsymbol {s}   \boldsymbol {s} ^T$ by $( \boldsymbol {s}   \boldsymbol {s} ^T +  \boldsymbol {s} ^*  \boldsymbol {s} ^{*T})/2$.

Applying *C*_*s*_ to the vector $\boldsymbol {v}$ then yields

20 $$ C_s \ \boldsymbol {v} =\frac{1}{2r} \int {\rm d} \boldsymbol {s}  P({\rm spike} |  \boldsymbol {s} ) P( \boldsymbol {s} ) \left(  \boldsymbol {s}   \boldsymbol {s} ^T \boldsymbol {v} +  \boldsymbol {s} ^*  \boldsymbol {s} ^{*T} \boldsymbol {v} \right) $$

Investigating the terms $ \boldsymbol {s}   \boldsymbol {s} ^T \boldsymbol {v}$ and $ \boldsymbol {s} ^*  \boldsymbol {s} ^{*T} \boldsymbol {v}$, we see that $ \boldsymbol {s} ^T \boldsymbol {v}$ and $ \boldsymbol {s} ^{*T} \boldsymbol {v}$ are equal in magnitude, but with opposite sign because of the mirror-image properties of $ \boldsymbol {s} $ and $ \boldsymbol {s} ^*$, see Fig. 3(b). The sum $ \boldsymbol {s}   \boldsymbol {s} ^T \boldsymbol {v} +  \boldsymbol {s} ^*  \boldsymbol {s} ^{*T} \boldsymbol {v}$ is therefore proportional to $ \boldsymbol {s}  -  \boldsymbol {s} ^* = 2(\boldsymbol {v}^T  \boldsymbol {s} )\boldsymbol {v}$. This vector is parallel to $\boldsymbol {v}$; the components orthogonal to $\boldsymbol {v}$ cancel out. Since this argument holds for every $ \boldsymbol {s} $, $C_s \boldsymbol {v}$ is proportional to $\boldsymbol {v}$, which is exactly the condition for $\boldsymbol {v}$ being an eigenvector of *C*_*s*_, $C_s \boldsymbol {v} = \lambda \boldsymbol {v}$.

We conclude that for a spherically symmetric stimulus distribution, an eigenvalue analysis of *C*_*s*_ yields a set of degenerate eigenvalues whose eigenvectors span the irrelevant space. For non-Gaussian stimuli, the numerical value of this eigenvalue baseline generally cannot be predicted and depends on the details of the nonlinearity *φ* within the LN model (Paninski 2003). For Gaussian stimuli, on the other hand, the baseline level of irrelevant eigenvalues is always equal to the prior variance (here fixed at 1) because individual stimulus components are independent and are thus not affected by changes of variance in other directions (Paninski 2003).

As a consequence, STC analysis can be applied to the general case of spherically symmetric stimulus distributions, not necessarily Gaussian. The relevant space, however, must now be identified as the one spanned by the eigenvectors whose eigenvalues depart from the baseline level of irrelevant eigenvalues. Note that in practical applications, the degeneracy of irrelevant eigenvalues is broken up by finite sampling effects and the eigenvalues scatter around the baseline. As explained in Section 5, statistical methods can be used to test whether the scatter is consistent with pure finite sampling effects of otherwise degenerate eigenvalues.

An alternative derivation of the main result of this subsection is provided by group theory. The argument is based in the fact that the firing probability $P({\rm spike} |  \boldsymbol {s} )$ remains invariant under any coordinate transformation operating only on the irrelevant subspace ${\cal K}_\perp$ and leaving the relevant subspace ${\cal K}$ unchanged. There are many such transformations, since the irrelevant subspace ${\cal K}_\perp$ is usually high-dimensional. All these coordinate transformations are called *symmetries* of the firing probability. The symmetries of the firing probability are also symmetries of the covariance matrix. As shown by many examples in quantum mechanics and solid state theory, the symmetry of a linear operator determines the degeneracy of its eigenvalues. Based on these ideas, in Appendix A, we rederive the main result of this subsection, using only symmetry arguments.

### Disambiguation of relevant and irrelevant spaces

Relevant directions are associated with eigenvalues that pop out as outliers of the baseline degenerate spectrum. Therefore, they can be easily identified only when the irrelevant space is high-dimensional, so that the spectrum reveals a highly degenerate eigenspace. Fortunately, in most practical cases the dimensionality of the relevant stimulus space is considerably smaller than the dimensionality of the complete stimulus space. We thus generally search for a small number of relevant stimulus directions immersed in a much larger stimulus space.

When working with small-dimensional stimulus spaces, however, it may not be apparent from the eigenvalue spectrum alone which eigenvectors belong to the relevant and which to the irrelevant space. In the scenario of Fig. 2(c2), for example, the stimulus space has only two dimensions, and STC analysis therefore just gives two (different) eigenvalues. The question then arises of how to test whether one of these directions—or more generally whether a given subspace with degenerate eigenvalue—actually corresponds to the irrelevant space.

As an example, imagine that the spectrum of eigenvalues reveals two (small-dimensional) degenerate subspaces, and we wish to determine which is relevant and which (if any) is irrelevant. As a first attempt, one could investigate the nonlinearity along one stimulus direction belonging to the hypothesized irrelevant space. The nonlinearity can be obtained by evaluating the probability that stimuli having a given projection value along the selected direction produce a spike, irrespective of its projection on the relevant space. One may call such test an evaluation of the “marginal nonlinearity”. In practical applications, probabilities are estimated from histograms (Chichilnisky 2001). When using Gaussian stimulus distributions, the marginal nonlinearity is (approximately) flat if the selected direction indeed belongs to the irrelevant space. For non-Gaussian prior distributions, however, the dependencies between different stimulus directions can cause a non-constant marginal nonlinearity even along irrelevant dimensions, and this method may thus not be conclusive.

As an alternative, we suggest to evaluate the “conditional nonlinearity”, obtained in the following way: For each direction of the hypothesized relevant space, choose a fixed target value (for example, zero) to condition the nonlinearity. Then compute the nonlinearity along a direction of the hypothesized irrelevant space by using only those stimuli whose corresponding projections on the putative relevant directions lie in a small window around the target values. The conditional nonlinearity is largely unaffected by the dependence between relevant and irrelevant stimulus directions; it should therefore be nearly flat if indeed the hypothesis about the irrelevant space was correct. The method works well as long as the putative relevant subspace is low dimensional and sufficient data are available. A disadvantage is that the amount of required data increases exponentially with the dimensionality of the relevant subspace.

Note that one can construct special scenarios where even the conditional nonlinearity does not disambiguate which subspace is relevant and which is irrelevant. One example is shown in Fig. 2(c3) where the two dimensions *x* and *y* are connected through the stimulus distribution by *x* ^2^ + *y* ^2^ = 1 and the spike probability is a function of *x* ^2^. Under the constraint of this particular stimulus distribution, this model cannot be distinguished from an equivalent model description where *y* is considered a relevant direction, with the spike probability a function of *y* ^2^ = 1 − *x* ^2^. More generally, the disambiguation based on conditional nonlinearities fails whenever the prior stimuli are sampled from the surface of a high-dimensional sphere (introducing a constraint that lets the square of one component be expressed in terms of the other components) and the spiking probability depends on a quadratic form of some or all of the relevant stimulus components. Identification of the actual relevant directions, defined by the nonlinearity (Fig. 2(a2)) independently of the applied stimulus distribution, then has to rely on other sources of information, for example, prior expectations about which stimulus components should be relevant (typically the expectation that the relevant subspace is small-dimensional) or additional experiments performed with a different stimulus distribution. It is interesting to note that STA analysis, naturally, suffers from the same ambiguity in the considered scenarios. When the nonlinearity depends on a quadratic form of the inputs, for example, when it is an even function, the STA must be identical to zero.

### Covariance analysis with or without subtracting the STA

Coming back to the geometrical derivation, note that the $\langle  \boldsymbol {s} \rangle\langle  \boldsymbol {s} \rangle^T$-term in Eq. (17) played essentially no role in the proof. We had shown that applying this term to any irrelevant direction $\boldsymbol {v}$ just yields zero; the derivation that $\boldsymbol {v}$ is an eigenvector of the STC matrix is thus valid also if this term is left out when calculating the STC matrix. It follows that the STC method works independently of whether the STA is subtracted from the spike-triggered ensemble or not, for example when computing the sample covariance matrix, Eq. (16); in both cases, all irrelevant directions yield degenerate eigenvalues (see also the first example below).

Furthermore, it also follows that the method works if the STA is projected out of the stimulus ensemble, so that only dimensions orthogonal to the STA are taken into account (Rust et al. 2004, 2005; Simoncelli et al. 2004; Schwartz et al. 2006). As the STA is part of the relevant subspace ${\cal K}$, projecting it out simply reduces the dimensionality of the relevant subspace by one and does not affect the irrelevant subspace. The complete relevant subspace may be reconstructed by combining the STA with the relevant directions obtained from the reduced STC analysis. This approach can be useful to avoid the scenario where a relevant direction might not be detected by STC analysis alone because it happens to have the same eigenvalue as the irrelevant directions.

### Examples

In this section, two examples are presented. The first one compares the results of covariance analysis with and without subtracting the STA in the spike-triggered covariance matrix, Eq. (16). The second one discusses the difference between Gaussian and non-Gaussian stimuli.

#### STC with and without subtracting the STA

**Fig. 4:**
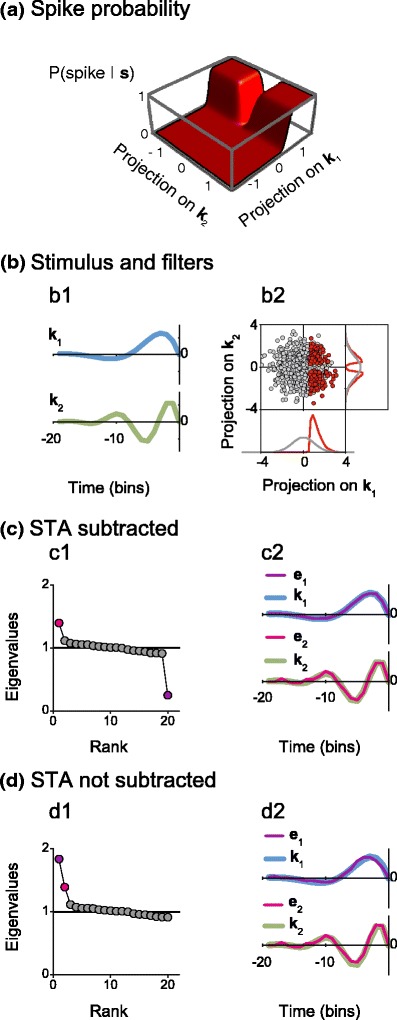
Covariance analysis can be carried out with (**c**) or without (**d**) subtracting the STA. (**a**) Spike probability in the relevant space: $ P({\rm spike} | \boldsymbol s ) = \{1 - \exp[-(\boldsymbol k_2^T \boldsymbol s )^2/0.05]\}/\{1 + \exp[- (\boldsymbol k _1^T \boldsymbol s  - 0.5) / 0.05]\} $. (**b1**) Relevant filters $\boldsymbol {k}_1$ and $\boldsymbol {k}_2$. (**b2**) Prior (*gray*) and spike-generating (*red*) stimuli in the subspace spanned by $\boldsymbol {k}_1$ and $\boldsymbol {k}_2$. The stimulus was Gaussian white noise with unit variance. (**c1**) and (**d1**) Eigenvalues of *C*_*s*_. The *black line* indicates the value 1. (**c2**) and (**d2**) Comparison between the relevant filters $\boldsymbol {k}_1$ and $\boldsymbol {k}_2$ and the eigenvectors $\boldsymbol {e}_1$ and $\boldsymbol {e}_2$ corresponding to the eigenvalues of matching color in (**c1**) and (**d1**). Number of analyzed spikes in each example: 5000

Covariance analysis can be equally performed with and without subtracting the STA in the calculation of the spike-triggered covariance matrix, as shown in Fig. 4.

In this example, there are two relevant directions, $\boldsymbol {k}_1$ and $\boldsymbol {k}_2$ (panel (b1)). The spiking probability (Fig. 4(a)) is highest for stimuli whose projection on $\boldsymbol {k}_1$ is large and whose projection on $\boldsymbol {k}_2$ is large in absolute value, as reflected by the distribution of spike-generating stimuli (panel (b2)). The STA is proportional to $\boldsymbol {k}_1$. Figure 4(c) shows the eigenvalues and eigenvectors obtained when diagonalizing the covariance matrix *C*_*s*_ with the STA subtracted. The largest eigenvalue (panel (c1)) represents the direction where the spike-generating stimuli have maximal variance, in this case, $\boldsymbol {k}_2$. The smallest eigenvalue corresponds to the direction with minimal variance: $\boldsymbol {k}_1$. The two relevant directions, $\boldsymbol {k}_1$ and $\boldsymbol {k}_2$, are accurately captured by the relevant eigenvectors $\boldsymbol {e}_1$ and $\boldsymbol {e}_2$, as shown in Fig. 4(c2).

In Fig. 4(d), we illustrate the diagonalization of the spike-triggered covariance matrix without subtracting the STA. The eigenvalues now represent the mean square projection of spike-generating stimuli along each direction. Two eigenvalues lie above the baseline level (panel (d1)). Although the eigenvalues are numerically different from those obtained in panel (c1), the eigenvectors coincide (compare panel (d2) with (c2)). The relevant filters, hence, can be obtained by diagonalizing *C*_*s*_ either with or without subtracting the STA.

#### Comparing Gaussian and non-Gaussian stimuli

**Fig. 5:**
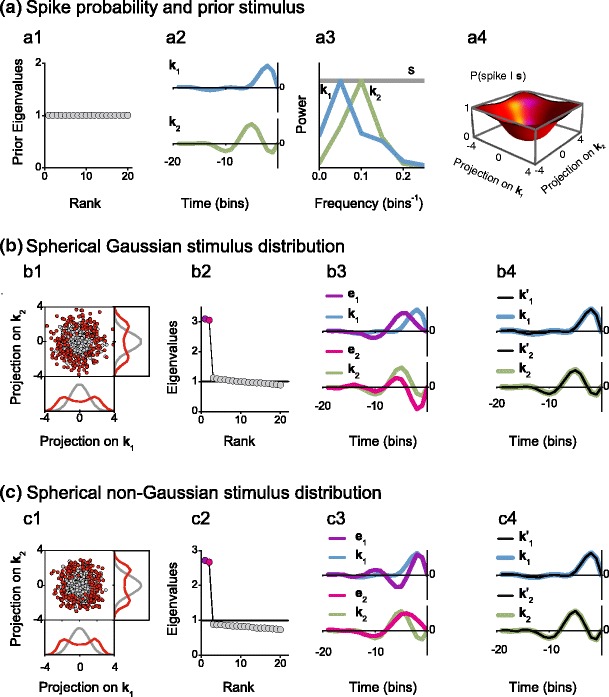
Covariance analysis using Gaussian (**b**) and spherical non-Gaussian (**c**) stimulus distributions. (**a1**) Prior eigenvalues. (**a2**) and (**a3**) Filters governing the firing probability in the time and frequency domains. (**a4**) Spike probability in the relevant space: $P({\rm spike} | \boldsymbol s) = (1 - \exp\{-[(\boldsymbol {k}_1^T \boldsymbol s / 2.2)^2 + (\boldsymbol {k}_2^T \boldsymbol s / 2.2)^2]\})^{4}$. (**b**) Gaussian prior stimulus. (**b1**) Prior (*gray*) and spike-generating (*red*) stimuli. (**b2**) Eigenvalues of *C*_*s*_. The *black line* indicates the value 1. (**b3**) Eigenvectors $\boldsymbol {e}_1$ and $\boldsymbol {e}_2$ corresponding to the eigenvalues of matching colors in b2 and comparison with the filters $\boldsymbol {k}_1$ and $\boldsymbol {k}_2$. (**b4**) Comparison between the filters $\boldsymbol {k}_1$ and $\boldsymbol {k}_2$ and their projections $\boldsymbol {k}_1^{\prime}$ and $\boldsymbol {k}_2^{\prime}$ on the space generated by $\boldsymbol {e}_1$ and $\boldsymbol {e}_2$. (**c**) Same as above, for non-Gaussian prior stimuli. The stimuli belong to the surface of a 20-dimensional sphere with unit variance along each component. Number of analyzed spikes in each example: 5000

In the example of Fig. 5, the difference between Gaussian and non-Gaussian prior stimuli is exemplified. Both applied stimulus distributions are spherically symmetric, and the eigenvalues of their prior covariance matrices are all equal to 1 (panel (a1)). The relevant space is spanned by the filters $\boldsymbol {k}_1$ and $\boldsymbol {k}_2$, and these two vectors differ in their shape (panel (a2)) and frequency content (panel (a3)). The firing probability (panel (a4)) has rotational symmetry in the relevant space. When the stimulus is Gaussian (Fig. 5(b)), all irrelevant eigenvalues cluster around unity (panel (b2)). In contrast, for non-Gaussian stimuli, irrelevant eigenvalues may cluster around some other value, here around a baseline of 0.8 (panel (c2)).

The fact that the baseline is below unity is actually a consequence of the shape of the prior stimulus distribution, which here has the form of a 20-dimensional spherical shell, and of the increased variance of the spike-triggered stimuli along the relevant stimulus components. Spikes only occur when the absolute value of the components along the relevant directions are large. Since the norm of each $ \boldsymbol {s} $ is fixed, vectors with large relevant components necessarily have small irrelevant components. The degenerate eigenvalues at 0.8 < 1 reflect the reduced variance along irrelevant directions. However, if the nonlinearity of the model *φ* were changed so that spikes were only triggered by stimuli having small components along the relevant directions, the fixed stimulus norm would force these stimuli to have large irrelevant components. The baseline of the irrelevant eigenvalues would then be above unity. This argument holds for a shell-like prior distribution; for a different stimulus, say one where the radial component of the prior distribution is sharply peaked at the origin, the relationship between the variance along the relevant directions and the baseline of the irrelevant eigenvalues may be inverted: Increased variance along relevant directions corresponds to a baseline above unity; decreased variance, to a baseline below unity.

In both scenarios of Fig. 5, the two relevant stimulus directions are identified by the two outliers of the spectrum (panels (b2) and (c2)). Note that the obtained relevant eigenvectors and the original filters of the model do not match in a one-by-one fashion. The two pairs of vectors, however, span the same space, since each filter $\boldsymbol {k}_m$ coincides with its projection $\boldsymbol {k}_m^\prime$ on the space generated by $\boldsymbol {e}_1$ and $\boldsymbol {e}_2$ (panels (b4) and (c4)). The identification of the relevant space rather than of the individual model filters is, in fact, all that one can expect from STC analysis; in the expression of the firing probability, Eq. (5), the individual filters $\boldsymbol {k}_m$ are not uniquely defined and could be exchanged for others that span the same relevant space, provided that the nonlinearity *φ* be appropriately adjusted. Thus, the expression of the firing probability used in Fig. 5 could have been formulated in a mathematically equivalent way, using a different pair of filters that span the same subspace.

In the present example, the identification of the subspace, but not the individual filters is particularly obvious because the firing probability has rotational symmetry in the two-dimensional relevant subspace. As discussed also in Appendix A, this symmetry leads to degenerate eigenvalues for the two relevant directions, as shown in panels (b2) and (c2). Therefore, the whole space generated by their linear combinations is an eigenspace of the covariance matrix.

## Extension to elliptic stimulus distributions

In this section, we generalize the previous results to the case of elliptic prior stimulus distributions. Elliptic distributions represent a special case of non-white stimulus distributions. Individual stimulus components are now correlated, and the prior covariance matrix *C*_*p*_ is no longer the unit matrix.

One way of dealing with an elliptic prior stimulus distribution when, in addition, the distribution is Gaussian has been pointed out by Bialek and de Ruyter van Steveninck (2005). When the eigenvalue analysis is carried out on the matrix Δ*C* = *C*_*s*_ − *C*_*p*_, relevant directions are marked by eigenvalues that deviate from the baseline of zero and are obtained from the corresponding eigenvectors after premultiplication with $C_p^{-1}$. The correction with $C_p^{-1}$ undoes the correlations that are induced by the prior stimulus distribution. However, this method requires a Gaussian distribution of stimuli. In the following, we aim at deriving an analogous procedure only relying on the elliptic symmetry of the prior stimulus distribution.

An elliptic stimulus distribution is one that can be made spherical by linearly rescaling the components of the stimulus along appropriately chosen *N* orthogonal axes. The procedure is the same as the one needed to transform an ellipsoid into a sphere: Each of the principal axes of the ellipsoid is divided by its length. Obtaining an extension of STC analysis is then straightforward: Transform stimuli so that they assume a spherical distribution, apply STC analysis to the transformed stimulus distribution, and then transform back the obtained relevant and irrelevant directions to the original stimulus space. We now go through this procedure step by step in order to arrive at a condensed prescription.

### Transforming to a spherical distribution

We first need to identify the principal axes of the prior stimulus distribution $P( \boldsymbol {s} )$. These are the eigenvectors of the prior covariance matrix *C*_*p*_. Because *C*_*p*_ is, like all covariance matrices, symmetric and positive-semidefinite, *C*_*p*_ can be transformed by an orthogonal transformation *O* into a diagonal matrix *D* with real-valued, non-negative diagonal elements,

21 $$ O^T \ C_p \ O = D. $$

Let us assume for the moment that all diagonal elements of *D* are larger than zero so that *D* has full rank. We can then calculate *D* ^1/2^ by taking the square root of each diagonal element of *D* and *D* ^− 1/2^ by in addition taking the inverse.

The prior distribution $P( \boldsymbol {s} )$ is called *elliptic* if it can be transformed into a spherical distribution by defining new rescaled coordinates

22 $$  \boldsymbol {s} ^\prime = D^{-1/2}\ O^T\  \boldsymbol {s} . $$

This transformation maps the original space of vectors $ \boldsymbol {s} $ to the *symmetric* space of vectors $ \boldsymbol {s} ^{prime}$. The matrices required to perform covariance analysis can also be transformed to the symmetric space. The transformed stimuli have a prior covariance matrix that is equal to the identity matrix

23 $$ C_p^\prime = \int \ {\rm d} \boldsymbol {s} ^{\prime} \ P( \boldsymbol {s} ^{\prime}) \  \boldsymbol {s} ^{\prime} { \boldsymbol {s} ^\prime}^T = \mathbb{I}. $$

The spike-triggered covariance matrix in the transformed stimulus space $C_s^\prime$ can simply be obtained by calculating the spike-triggered covariance matrix in the original space and then transforming appropriately,

24 $$ C_s^\prime = D^{-1/2} \ O^T \ C_s \ O \ D^{-1/2}. $$

Equation (24) follows from the fact that

25 $$  \boldsymbol {s} ^\prime \ { \boldsymbol {s} ^\prime}^T = D^{-1/2} \ O^T \  \boldsymbol {s}  \  \boldsymbol {s} ^T \ O \ D^{-1/2} . $$

In the symmetric space, the stimulus distribution is spherical, so the results of Section 3 are applicable. The irrelevant directions can be obtained as the eigenvectors of $C_s^\prime$ whose eigenvalues constitute the baseline degenerate spectrum. The relevant space is the orthogonal complement of the irrelevant space.

We now return the relevant and irrelevant directions back to the original space. In order to obtain the transformation rules for the relevant directions, care has to be taken to preserve the scalar products. The conditional firing probability given by Eq. (5) must remain unchanged when transforming from $ \boldsymbol {s} ^{\prime}$ to $ \boldsymbol {s} $. We therefore require $\varphi( \boldsymbol {s} ^{\prime}) = \varphi( \boldsymbol {s} )$. This condition is fulfilled if relevant directions in the transformed space, $\boldsymbol {w}^\prime$, are connected to relevant directions of the original space, $\boldsymbol {w}$, through the condition

26 $${\boldsymbol {w}^{\prime}}^T \  \boldsymbol {s} ^{\prime} = \boldsymbol {w}^T \  \boldsymbol {s}  $$

for all original stimuli $ \boldsymbol {s} $ and their transformed versions $ \boldsymbol {s} ^\prime$. The transformation properties of the relevant directions $\boldsymbol {w}$ are then defined in terms of their scalar products to stimulus vectors. In mathematical terms, this means that the relevant directions $\boldsymbol {w}$ do not transform as the original vectors $ \boldsymbol {s} $, but as dual vectors (in physics, the terminology of covariant and contravariant vectors is also used). Hence, the transformation rule for relevant directions is

27 $$ \boldsymbol {w}^{\prime} = D^{1/2} \ O^T \ \boldsymbol {w}, $$

and the backward transformation is

28 $$ \boldsymbol {w} = O \ D^{-1/2} \ \boldsymbol {w}^\prime . $$

Note that Eq. (27) is not equivalent to Eq. (22). Also note that the obtained $\boldsymbol {w}$ are not necessarily orthogonal to each other, in contrast to the eigenvectors that are obtained for spherically symmetric stimulus distributions. However, the set of relevant directions is still linearly independent and thus spans a relevant subspace of the same dimensionality as the relevant directions $\boldsymbol {w}^\prime$ in the symmetric space.

For completeness, we also provide the transformation properties of irrelevant directions. In the symmetric space, irrelevant directions are orthogonal to relevant ones, since relevant and irrelevant directions are eigenvectors of a symmetric matrix. In the original space, irrelevant directions must still be orthogonal to relevant directions: this orthogonality is what defines them, because it ensures that they do not contribute to any of the scalar products $\boldsymbol {k}_m^T  \boldsymbol {s} $ in Eq. (5). Orthogonality is guaranteed if irrelevant directions are transformed with the same prescription as the stimulus vectors. Thus, if $\boldsymbol {v}$ is an irrelevant vector, then

29 $$ \boldsymbol {v}^{\prime} = D^{-1/2}\ O^T\ \boldsymbol {v}, $$

and backward,

30 $$ \boldsymbol {v} = O \ D^{1/2} \ \boldsymbol {v}^\prime . $$

Note that Eq. (29) is equivalent to the transformation of stimulus vectors, Eq. (22), but not to the transformation of relevant stimulus directions, Eq. (27).

We now summarize the procedure of STC analysis for elliptic stimulus distributions:

1. Calculate the spike-triggered covariance matrix *C*_*s*_ with the original stimuli.
2. Obtain the transformed STC matrix $C_s^\prime$ using Eq. (24).
3. Obtain the transformed relevant directions $\boldsymbol {w}^\prime$ and irrelevant directions $\boldsymbol {v}^\prime$ from an eigenvalue analysis of $C_s^\prime$.
4. Obtain the original relevant directions $\boldsymbol {w}$ and irrelevant directions $\boldsymbol {v}$ by transforming back with Eqs. (28) and (30), respectively.

In passing, we mention that when the spike probability *φ* contains a single relevant direction $\boldsymbol {k}_1$, the transformation to the symmetric space is also applicable to the calculation of the STA. As shown in Appendix B, this procedure leads to the well-known recipe of estimating the single relevant direction by premultiplying the STA by the inverse of the prior covariance matrix: $\boldsymbol {k}_1 \propto C_p^{-1} \langle  \boldsymbol {s}  \rangle$ (Theunissen et al. 2001; Paninski 2003; Schwartz et al. 2006).

### STC analysis directly in the original stimulus space

For convenience, we now reformulate the whole procedure using only quantities defined in the original space. Operationally, this can spare us from the need to transform forth and back to the symmetric space. We first note that the two matrices $C_p^{-1} C_s$ and $C_s C_p^{-1}$ both have the same eigenvalue spectrum as $C_s^\prime$. In technical terms, they are both similar matrices to $C_s^\prime$: If *P* is the change-of-base matrix needed to transform relevant directions to the symmetric space (*P* = *D* ^1/2^ *O* ^*T*^, as stated in Eq. (27)), then, following Eq. (24), $C_p^{-1} C_s$ and $C_s^\prime$ are related by a similarity transformation: $C_p^{-1} C_s = P^{-1} C_s^\prime P$. Similar matrices share the same eigenvalues and have related eigenvectors: If in the symmetric space $\boldsymbol {w}^\prime$ is a relevant eigenvector of $C_s^\prime$ with eigenvalue *λ*, then in the original space, $\boldsymbol {w} = P^{-1} \boldsymbol {w}^\prime = O D^{-1/2} \boldsymbol {w}^\prime$, as in Eq. (28), is an eigenvector of $C_p^{-1} C_s$ with eigenvalue *λ*. Thus, relevant directions $\boldsymbol {w}$ can be identified from an eigenvalue analysis of $C_p^{-1} C_s$.

In the same way, if *Q* transforms irrelevant directions (that is, *Q* = *D* ^− 1/2^ *O* ^*T*^, as stated in Eq. (29)), then $C_s C_p^{-1} = Q^{-1} C_s^\prime Q$, which means that $C_s C_p^{-1}$ and $C_s^\prime$ share the same eigenvalues and have related eigenvectors. Thus, all irrelevant directions are eigenvectors of $C_s C_p^{-1}$ with degenerate eigenvalues. Note that $C_p^{-1} C_s$ and $C_s C_p^{-1}$ are generally not symmetric, and thus the eigenvectors of $C_p^{-1} C_s$ and of $C_s C_p^{-1}$, respectively, do not form orthogonal sets. Yet, each set of eigenvectors still provides a basis for the stimulus space because the eigenvectors of $C_s^\prime$ provide a basis and the transformations *P* and *Q* both have full rank.

Furthermore, relevant and irrelevant directions remain orthogonal to each other. To see this, note that the two matrices $C_p^{-1} C_s$ and $C_s C_p^{-1}$ are adjoint matrices, since they are real-valued and $(C_p^{-1} C_s)^T = C_s C_p^{-1}$. Adjoint matrices have the same set of eigenvalues, and moreover, their eigenvectors form dual bases. Thus, if $\boldsymbol {w}$ is an eigenvector of $C_p^{-1} C_s$ with eigenvalue *λ* and $\boldsymbol {v}$ is an eigenvector of $C_s C_p^{-1}$ with eigenvalue $\mu \ne \lambda$, then $\boldsymbol {v} \perp \boldsymbol {w}$. Therefore, relevant directions are confirmed to be perpendicular to irrelevant directions.

In summary, the problem of identifying relevant and irrelevant directions for general elliptic stimulus distributions may be entirely solved in the original space. Relevant directions are obtained as eigenvectors of $C_p^{-1} C_s$ corresponding to eigenvalues that differ from the degenerate baseline level. Irrelevant directions can be obtained from the orthogonal complement or as the eigenvectors of $C_s C_P^{-1}$ corresponding to eigenvalues of the degenerate baseline level. The fact that we need two different matrices, $C_p^{-1} C_s$ and $C_s C_p^{-1}$, reflects the different transformation properties of relevant and irrelevant directions. Using the matrices $C_p^{-1} C_s$ and $C_s C_P^{-1}$ serves as an alternative to the eigenvalue analysis of the transformed STC matrix $C_s^\prime$, Eq. (24), and then transforming the obtained eigenvectors according to Eqs. (28) and (30). In fact, these two methods yield identical eigenvalue spectra and (transformed) eigenvectors. Note that all derivations above still work if *C*_*s*_ is calculated without subtracting the STA.

### Regularization

So far, we have assumed that the prior covariance matrix *C*_*p*_ has full rank so that it can be inverted. However, if *D* has one or more vanishing diagonal elements, Eq. (22) is ill-defined. This happens when one or more stimulus directions have zero variance, so the prior stimuli do not cover all dimensions of the stimulus space. A typical example is given by low-pass filtered stimuli sampled at high frequency. If the prior stimulus lacks one or more dimensions, there is no way to extract information about the firing probability in the missing dimensions. The best we can do is estimate the filters without these dimensions. There are two ways to proceed. One is to simply eliminate the missing dimensions, that is, to work with a rectangular *D* matrix (more rows than columns). The transformation matrix *P* is therefore also rectangular, and the symmetric space has a lower dimensionality than the original space. The analysis can be carried out as before, only that when returning back to the original space, the number of relevant plus irrelevant directions is smaller than the dimensionality of the original space. The other alternative is to employ the full *D* matrix, but when inverting it, to set the infinite-valued diagonal elements of *D* ^− 1^ equal to zero. The matrix $C_p^{-1} = O D^{-1} O^T$ is then defined as the pseudo inverse of *C*_*p*_.

This regularization procedure is also advised when some of the eigenvalues of *C*_*p*_ are not necessarily zero, but much smaller than others. The corresponding dimensions are not well represented in the prior stimulus distribution and should thus be eliminated in the analysis to avoid noise amplification when inverting *C*_*p*_. Noise comes from limited sampling. Setting the diagonal elements of *D* ^− 1^ to zero when, for example, the corresponding diagonal elements of *D* are smaller than a certain fraction of the maximal eigenvalue (say 5 %) is a simple, yet effective way to regularize the prior stimulus distribution (Touryan et al. 2005; Felsen et al. 2005).

### Examples of covariance analysis with elliptic stimulus distributions

Here we present two examples where the prior stimulus distribution is elliptic. The first one is a Gaussian stimulus, for which different dimensions are independent from one another. The second one is a hollow-ellipsoid-like stimulus distribution, where components are coupled. In both cases, we employ the same nonlinearity and relevant filters as in Fig. 5. Our aim is to compare the results obtained by diagonalizing $C_p^{-1} C_s$ with those of Δ*C* = *C*_*s*_ − *C*_*p*_. As expected, the two methods are equivalent when the stimulus is Gaussian, but produce different results when applied to non-Gaussian elliptic stimulus distributions.

#### Example with a Gaussian elliptic stimulus distribution

**Fig. 6:**
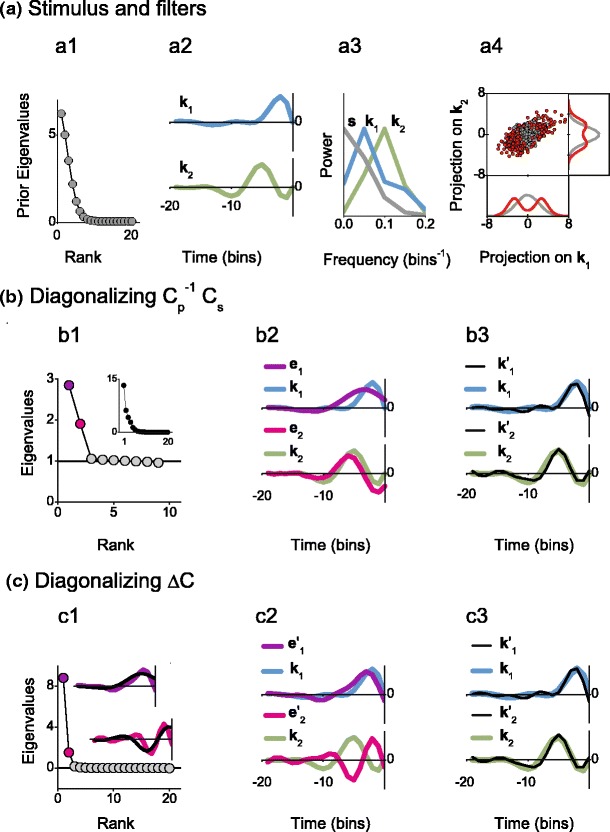
Covariance analysis with elliptic Gaussian stimulus distributions. (**a1**) Spectrum of prior eigenvalues. The prior stimulus was constructed in Fourier space. The real and imaginary components of each frequency *ν* were drawn from a Gaussian distribution of zero mean and standard deviation ${\rm std}(\nu) \propto \exp\left\{ - \nu^2 / [2 \cdot (0.075\ {\rm bins}^{-1})^2] \right\} + 0.1$ and then transformed back to the time domain and normalized to unit variance. (**a2**) and (**a3**) Relevant filters, displayed in the time and frequency domains. (**a4**) Prior (*gray*) and spike-generating (*red*) stimuli. The firing probability for this example was the same as the one used in Fig. 5. (**b1**) Spectrum of eigenvalues of $C_p^{-1}C_s$. Stimulus dimensions whose variance was less than 1.5 % of the maximal variance were projected out before the analysis. The *black line* indicates the value 1. For comparison, the inset shows the eigenvalues of *C*_*s*_. (**b2**) Comparison of the filters $\boldsymbol {k}_1$ and $\boldsymbol {k}_2$ to the eigenvectors $\boldsymbol {e}_1$ and $\boldsymbol {e}_2$ corresponding to the eigenvalues of matching colors in (**b1**). (**b3**) Comparison of the filters $\boldsymbol {k}_1$ and $\boldsymbol {k}_2$ to their projections $\boldsymbol {k}_1^\prime$ and $\boldsymbol {k}_2^\prime$ on the space generated by $\boldsymbol {e}_1$ and $\boldsymbol {e}_2$. (**c1**) Spectrum of eigenvalues of Δ*C*. The *black line* indicates the value 0. In the insets, the raw eigenvectors $\boldsymbol {e}_1$ and $\boldsymbol {e}_2$ are shown (*black lines*), together with their corrected versions $\boldsymbol {e}^\prime_1$ and $\boldsymbol {e}^\prime_2$ (*colored lines*). (**c2**) Comparison of the filters $\boldsymbol {k}_1$ and $\boldsymbol {k}_2$ to the corrected eigenvectors $\boldsymbol {e}^{\prime}_1$ and $\boldsymbol {e}^{\prime}_2$. (**c3**) Comparison of the filters $\boldsymbol {k}_1$ and $\boldsymbol {k}_2$ to their projections $\boldsymbol {k}_1^{\prime}$ and $\boldsymbol {k}_2^{\prime}$ on the space generated by $\boldsymbol {e}^{\prime}_1$ and $\boldsymbol {e}^{\prime}_2$. Number of analyzed spikes in each example: 5000

Figure 6 shows the diagonalization of $C_p^{-1} C_s$ and Δ*C* when the stimulus distribution is elliptic and Gaussian. An elliptic prior stimulus distribution gives rise to a non-uniform spectrum of prior eigenvalues (panel (a1)). We choose an example where the filters $\boldsymbol {k}_1$ and $\boldsymbol {k}_2$ have different temporal (panel (a2)) and frequency (panel (a3)) characteristics. Specifically, $\boldsymbol {k}_1$ has more power at lower frequencies compared to $\boldsymbol {k}_2$. Since the prior stimulus has an exponentially decaying spectrum, its variance in the direction $\boldsymbol {k}_1$ is larger than in the direction $\boldsymbol {k}_2$. Thus, the prior stimuli (gray dots) occupy an elongated region in stimulus space (panel (a4)). The firing probability is spherically symmetric in the subspace spanned by $\boldsymbol {k}_1$ and $\boldsymbol {k}_2$. Hence, the ratio of the density of red and gray dots in panel (a4) has circular contour lines. The transformation to the symmetric space contracts the elongated directions, resulting in a spherical prior distribution. As a consequence, the firing probability no longer looks spherically symmetric in the space spanned by $\boldsymbol {k}_1^{\prime}$ and $\boldsymbol {k}_2^{\prime}$. Due to this anisotropy, the degeneracy of the two relevant eigenvalues of $C_p^{-1} C_s$ is broken (panel (b1)).

In Fig. 6(b1), the total number of eigenvalues (9) is smaller than the dimensionality of the stimulus space (20). In this example, the prior stimulus has a small variance in 11 stimulus dimensions. To avoid noise amplification, the 11 sub-represented dimensions were eliminated before starting the analysis. The alternative strategy would have been to work with the pseudoinverse $C_p^{-1}$. In that case, the spectrum of $C_p^{-1} C_s$ would still have shown 20 eigenvalues, but 11 of them would have corresponded to the regularized directions and would therefore have been equal to zero.

When diagonalizing $C_p^{-1} C_s$, two eigenvalues deviate noticeably from unity (panel (b1)). For comparison, the eigenvalue spectrum of *C*_*s*_ itself is shown as an inset of panel (b1). This spectrum is similar to the one of *C*_*p*_, reflecting the fact that the spike-triggered stimulus distribution is strongly affected by the shape of the prior distribution. The eigenvectors $\boldsymbol {e}_1$ and $\boldsymbol {e}_2$ for the distinct eigenvalues of $C_p^{-1} C_s$ correspond to the elongated direction in panel (a4) (largest eigenvalue) and the perpendicular direction (second largest eigenvalue), respectively. The eigenvectors $\boldsymbol {e}_1$ and $\boldsymbol {e}_2$ are linear combinations of the filters $\boldsymbol {k}_1$ and $\boldsymbol {k}_2$, but they do not coincide with them (panel (b2)). The filters $\boldsymbol {k}_1$ and $\boldsymbol {k}_2$, however, coincide with their projections $\boldsymbol {k}_1^{\prime}$ and $\boldsymbol {k}_2^{\prime}$ on the space spanned by $\boldsymbol {e}_1$ and $\boldsymbol {e}_2$ (panel (b3)). This means that $\boldsymbol {k}_1$ and $\boldsymbol {k}_2$ span the same relevant space as $\boldsymbol {e}_1$ and $\boldsymbol {e}_2$.

In Fig. 6(c), the results of diagonalizing Δ*C* are displayed. Two eigenvalues are clearly above zero (panel (c1)). The associated eigenvectors $\boldsymbol {e}_1$ and $\boldsymbol {e}_2$ have a large fraction of their power in the low-frequency range, contaminated by the most represented direction in the prior stimulus. Consequently, they do not generate the same subspace as the filters $\boldsymbol {k}_1$ and $\boldsymbol {k}_2$. In order to correct for the ellipticity of the prior stimulus distribution, the eigenvectors must be premultiplied by $C_p^{-1}$ (Bialek and de Ruyter van Steveninck 2005), thus defining the corrected relevant eigenvectors $\boldsymbol {e}^{\prime}_1$ and $\boldsymbol {e}^{\prime}_2$. A comparison between the original eigenvectors $\boldsymbol {e}_1$ and $\boldsymbol {e}_2$ and their corrected versions $\boldsymbol {e}^{\prime}_1$ and $\boldsymbol {e}^{\prime}_2$ is shown in the insets of panel (c1). The correction procedure diminishes the low-frequency content of the filters. Although the corrected eigenvectors do not coincide with the individual filters $\boldsymbol {k}_1$ and $\boldsymbol {k}_2$ (see panel (c2)), they generate the same subspace, as evidenced by the excellent match between the filters $\boldsymbol {k}_i$ and their projections $\boldsymbol {k}_i^{\prime}$ on the space generated by $\boldsymbol {e}_1^\prime$ and $\boldsymbol {e}_2^\prime$.

#### Example with a non-Gaussian elliptic stimulus distribution

**Fig. 7:**
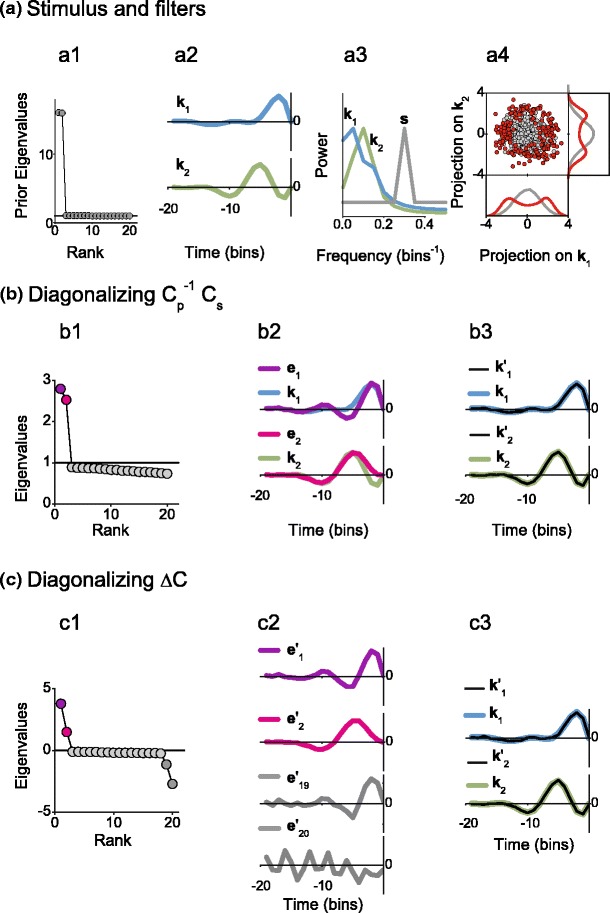
Covariance analysis with elliptic non-Gaussian stimulus distributions. (**a1**) Spectrum of prior eigenvalues. (**a2**) and (**a3**) Relevant filters, in the time (**a2**) and frequency (**a3**) domains. (**a4**) Prior (*gray*) and spike-generating (*red*) stimuli. The firing probability for this example was the same as the one used in Fig. 5. (**b1**) Spectrum of eigenvalues of $C_p^{-1}C_s$. The *black line* indicates the value 1. (**b2**) Comparison of the filters $\boldsymbol {k}_1$ and $\boldsymbol {k}_2$ to the eigenvectors $\boldsymbol {e}_1$ and $\boldsymbol {e}_2$ corresponding to the eigenvalues of matching colors in (**b1**). (**b3**) Comparison of the filters $\boldsymbol {k}_1$ and $\boldsymbol {k}_2$ to their projections $\boldsymbol {k}_1^{\prime}$ and $\boldsymbol {k}_2^{\prime}$ on the space generated by $\boldsymbol {e}_1$ and $\boldsymbol {e}_2$. (**c1**) Spectrum of eigenvalues of Δ*C*. The *black line* indicates the value 0. (**c2**) Corrected eigenvectors corresponding to the eigenvalues of matching color in (**c1**). (**c3**) Comparison of the filters $\boldsymbol {k}_1$ and $\boldsymbol {k}_2$ to their projections $\boldsymbol {k}_1^{\prime}$ and $\boldsymbol {k}_2^{\prime}$ on the space generated by $\boldsymbol {e}^{\prime}_1, \boldsymbol {e}^{\prime}_2$, $\boldsymbol {e}^{\prime}_{19}$, and $\boldsymbol {e}^{\prime}_{20}$. Number of analyzed spikes in each example: 5000

In Fig. 7, covariance analysis is performed on stimuli consisting of a collection of vectors lying on the surface of a 20-dimensional ellipsoid. Each vector is initially constructed from a spherical Gaussian distribution and then normalized to have unit length. Then, one Fourier component is multiplied by a factor of 4. Thus, the final prior stimuli lie on the surface of a 20-dimensional ellipsoid, with a variance of 16 in two Fourier components (two components, corresponding to sine and cosine phases at this frequency) and unit variance in the remaining directions (panel (a1)).

The two relevant filters (panels (a2) and (a3)) and the spiking probability are identical to those used in previous examples (Figs. 5 and 6). In the present case, however, the prior stimulus has approximately constant variance throughout the frequency range covered by the two filters (panel (a3)). Thus, the relevant space is almost fully included in the subspace spanned by the short directions of the prior stimulus and is perpendicular to the two elongated directions. Consequently, the prior stimulus has approximately the same variance in the directions of the two relevant filters, as seen by the circular symmetry of the gray dots in panel (a4). Thus, when transforming to the symmetric space, the two relevant directions are scaled by the same factor. The spectrum of $C_p^{-1} C_s$, hence, shows two roughly degenerate eigenvalues (panel (b1)). The associated eigenvectors $\boldsymbol {e}_1$ and $\boldsymbol {e}_2$ do not necessarily coincide with the filters $\boldsymbol {k}_1$ and $\boldsymbol {k}_2$ (panel (b2)). However, $\boldsymbol {k}_1$ and $\boldsymbol {k}_2$ perfectly coincide with their projections $\boldsymbol {k}_1^{\prime}$ and $\boldsymbol {k}_2^{\prime}$ on the space spanned by $\boldsymbol {e}_1$ and $\boldsymbol {e}_2$, verifying that the eigenvectors of $C_p^{-1} C_s$ span the same space as the filters.

The eigenvalue spectrum of Δ*C* has four clear outliers (panel (c1)). In this example, the two largest eigenvalues correspond to eigenvectors that, when corrected with $C_p^{-1}$, span the space generated by the filters $\boldsymbol {k}_1$ and $\boldsymbol {k}_2$. The smallest two eigenvalues, however, are spurious.

If a stimulus is Gaussian, different components are independent from one another. Hence, the distribution of spike-triggered stimuli along irrelevant directions coincides with the prior distribution. In this context, it makes sense to compensate for the stimulus ellipticity by subtracting *C*_*p*_ from *C*_*s*_: The variance of Δ*C* vanishes along irrelevant directions. For non-Gaussian stimuli as the one of Fig. 7, however, different components are not independent from one another. Hence, the variance of the spike-triggered stimuli does not coincide with the prior variance along irrelevant directions. The subtraction *C*_*s*_ − *C*_*p*_ is therefore not able to counteract the elliptic nature of the stimulus distribution, and now Δ*C* may have non-vanishing variance along irrelevant directions. Since the magnitude of the residual variance depends on the magnitude of the prior variance, the degeneracy of the irrelevant directions is broken. In the example of Fig. 7, we chose a prior stimulus whose spectrum is rather peculiar, and therefore, the loss of degeneracy of the irrelevant directions is very pronounced. More typically, the spectrum of the prior stimulus may decay in a fairly continuous way. The eigenvalues of Δ*C* associated with irrelevant directions, hence, also decay continuously. Whether they appear as clear outliers, or just as a weirdly shaped spectrum, depends on the exact numerical value of the prior spectrum, on the nonlinearity *φ*, and on the amount of collected data. In any case, they are not degenerate.

## Significance testing

**Fig. 8:**
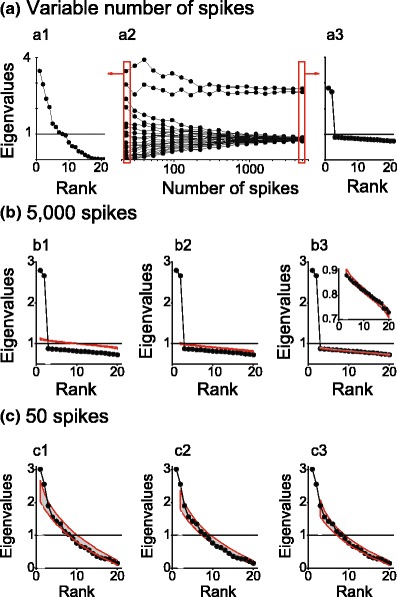
Significance testing of relevant stimulus directions. (**a**) Effect of finite sampling on the eigenvalue spectrum. (**a1**) The spectrum of ranked eigenvalues shows a smooth decay when only few spikes are analyzed (23 spikes). (**a2**) As more and more spikes are included in the analysis, eigenvalues of irrelevant directions converge and eigenvalues of relevant directions become distinctly separated from the baseline. (**a3**) In the limit of large data sets (here 5,000 spikes), the spectrum arrives at a clear distinction between relevant and irrelevant eigenvalues. (**b**) and (**c**) Comparison of the eigenvalues obtained from the actual spikes (*black circles*) to 95 % confidence intervals (gray area delimited by *red lines*) obtained from randomly rotating spike-triggered stimuli in the hypothesized irrelevant space. (**b**) 5,000 analyzed spikes. (**c**) 50 analyzed spikes. (**b1**) and (**c1**) Rotations are performed in the full 20-dimensional space. Eigenvalues lie outside the confidence interval. (**b2**) and (**c2**) After the stimulus component in the direction of the eigenvector of the first eigenvalue is projected out, rotations are performed in the remaining 19-dimensional space. The second eigenvalue still lies outside the confidence interval. (**b3**) and (**c3**) When the stimulus components in the directions of the first two eigenvectors are projected out, rotations are performed in the remaining 18-dimensional space. Now all remaining eigenvalues fall inside the confidence interval, so the corresponding stimulus space retains a degree of spherical symmetry that is compatible with the irrelevant space

The identification of the relevant stimulus space is based on recognizing degenerate eigenvalues. Due to finite sampling, however, the spectrum of eigenvalues for irrelevant directions is never perfectly degenerate, but rather shows some scatter around the value that would be expected in the limit of infinite amounts of data. The question then arises whether the scatter observed in a given spectrum represents true differences in variance along relevant directions or instead results from statistical fluctuations along irrelevant directions. Figure 8 displays the spectra of the same model as in Fig. 5(c), but now varying the number of spikes included in the analysis. In panel (a1), only 23 spikes are employed, and there, it is not possible to determine by naked eye which eigenvalues belong to the degenerate baseline level and which are the outliers. In panel (a3), on the other hand, with 5,000 spikes, the task appears trivial. To see how statistical fluctuations in the spectrum depend on the amount of available data, a useful visualization is to plot the evolution of the spectrum with increasing number of analyzed spikes (Agüera y Arcas and Fairhall 2003; Agüera y Arcas et al. 2003), as done in panel (a2). The eigenvalues corresponding to the irrelevant subspace converge progressively, whereas the ones associated with relevant directions branch off and settle at a distinct level. For a more systematic analysis of whether individual eigenvalues indicate relevant directions or not, we need a statistical test for the significance of deviations from degeneracy.

In the case of a Gaussian prior stimulus distribution, such a test is typically performed by randomly shifting the spike times and thereby generating artificial spike trains that, by construction, contain no relevant directions (Touryan et al. 2002; Rust et al. 2005; Schwartz et al. 2006). Hence, the resulting spike trains generate eigenvalue spectra that deviate from degeneracy only through finite-sampling effects that result from the number of analyzed spikes. The actual spectrum is then compared to the range of values obtained from the randomly shifted spike trains. Only outliers that significantly deviate from the resampled range qualify as eigenvalues associated with relevant directions.

The procedure has to be performed in a nested fashion because the finite-size effects depend on the dimensionality of the investigated stimulus subspace. First, the full stimulus space is tested. If its eigenvalue spectrum is found to be inconsistent with having no relevant directions, the stimulus direction corresponding to the eigenvalue that deviates most from unity is identified as a relevant direction and projected out from all stimuli. Next, the analysis is repeated in the reduced stimulus space. The procedure is iterated until the remaining eigenvalue spectrum is consistent with no further relevant directions.

For the case of a non-Gaussian stimulus distribution, this procedure is not directly applicable. The reason is, again, that relevant and irrelevant stimulus directions are not independent. Randomly shifting spike times creates a new ensemble of stimulus segments whose statistics are then compared to the spike-triggered stimulus ensemble. However, the statistics of such an artificial stimulus ensemble differ from the spike-triggered ensemble even along irrelevant directions. The simplest example is that the variance along irrelevant directions within the spike-triggered stimulus ensemble differs from unity (as seen in the baseline level of eigenvalues in Fig. 5(c2)), but for a random stimulus ensemble, this variance is equal to the prior variance, set to unity.

We therefore use a different resampling strategy to test whether the eigenvalue spectrum of a candidate subspace is consistent with a spectrum expected from an irrelevant space. To do so, we note that the distribution of spike-triggered stimuli retains the spherical symmetry inside the irrelevant subspace. If the candidate subspace is indeed the irrelevant subspace, the distribution of projections of the spike-triggered stimuli on the proposed subspace must be spherically symmetric, at least inasmuch as can be expected for the analyzed amounts of data. The null-hypothesis that we aim to test is thus whether the observed distribution of eigenvalues is consistent with spherical symmetry within the candidate subspace, given finite sampling. Therefore, resampling is carried out by randomly rotating each spike-triggered stimulus within this subspace. The random rotation of each spike-triggered stimulus can easily be obtained by taking the projection of the stimulus onto the candidate subspace, computing its vector length, and replacing the projection by a vector with the same length in a random direction within the candidate subspace. Practically, the random direction can be obtained, for example, by randomly drawing vector components from a Gaussian distribution and then normalizing the obtained random vector.

The resulting resampled stimulus distribution is, by construction, spherically symmetric in the investigated subspace, but retains the original distribution of absolute values. Therefore, if the candidate subspace is indeed an irrelevant subspace, then the eigenvalues of the resampled stimuli necessarily scatter around the same baseline level as the eigenvalues of the actual spike-triggered stimuli. We thus perform STC analysis on the set of rotated spike-triggered stimuli and repeat this procedure many times in order to determine the mean value of each of the ranked eigenvalues as well as confidence intervals.

Just like the resampling procedure that is based on shifting spike times, this analysis is performed in a nested fashion. The procedure is illustrated in Fig. 8(b) and (c) for the model used in Fig. 5(c) with a spherically symmetric stimulus distribution on the surface of a 20-dimensional sphere and two relevant directions. In the first round, all spike-triggered stimuli are rotated in the full *N*-dimensional stimulus space, and we test the null-hypothesis that there are no relevant directions, so the entire spike-triggered stimulus distribution is spherically symmetric. If the eigenvalues do not lie within the pre-specified confidence limits, say 95 % confidence intervals, the hypothesis is rejected, as is the case of panels (b1) and (c1). The eigenvector whose eigenvalue deviates most from the confidence interval is then identified as a relevant direction, and it is projected out from all spike-triggered stimuli for further significance testing. In the second round, we test the null-hypothesis that there are no relevant directions in the remaining stimulus space, based on the remaining *N* − 1 eigenvalues. Now, stimulus lengths are calculated in the remaining (*N* − 1)-dimensional subspace, and eigenvalue spectra of randomly rotated vectors are obtained from random vectors with the correct vector lengths in an (*N* − 1)-dimensional space. The procedure is iterated until the remaining eigenvalues lie within the specified confidence intervals. Figure 8(c) shows that the method correctly identifies two relevant filters in our example, even when using as few as 50 spikes and having no obvious degenerate baseline.

The significance test for the sphericity of stimulus distributions can easily be extended to elliptic distributions. The null-hypothesis should now state that the distribution of spike-triggered stimuli in the investigated subspace displays the same elliptic symmetry as the prior distribution. To use the resampling procedure, one can thus simply determine the stimulus length of a spike-triggered stimulus after applying the whitening transformation, Eq. (22), and then perform the rotation, the subsequent eigenvalue analysis, and the elimination of identified relevant directions in the transformed, spherically symmetric space.

## Discussion

Spike-triggered covariance analysis is generally used for identifying multiple relevant dimensions from Gaussian stimuli by finding differences in variance between the prior and the spike-triggered stimulus distributions. It has been noted that in this form, the analysis is not applicable to non-Gaussian stimuli (Paninski 2003; Simoncelli et al. 2004; Schwartz et al. 2006), in contrast to spike-triggered average analysis. Here, we have provided a geometric picture of STC analysis and have thereby shown that STC analysis is applicable to general spherically symmetric distributions when the criterion for identifying relevant directions is modified. The modified criterion consists of detecting eigenvalues of the STC matrix that differ from the common baseline of degenerate eigenvalues, even if this baseline does not correspond to the variance of the prior distribution. Moreover, we have shown that the new approach can also be extended to elliptic stimulus distributions. We thus conclude that the consistency of STC analysis requires special symmetries in the prior stimulus distribution (spherical, or more generally, elliptic). Gaussianity, instead, is not indispensable.

### Non-Gaussian stimuli in practice

Non-Gaussian spherical or elliptic stimulus distributions are, of course, not nearly as frequently encountered in experimental situations as Gaussian distributions, primarily because time series of Gaussian stimuli $ \boldsymbol {s} $ can be obtained in a continuous fashion by drawing new stimulus components *s*(*t*) from a Gaussian distribution. No such continuous generation of stimuli with a spherical, non-Gaussian distribution is possible. Application of the extended method may thus become useful when experiments are performed with stimuli that do not contain temporal dimensions, for example, when the components of $ \boldsymbol {s} $ represent spatial stimulus elements (cf. Fig. 1(b)). In this case, general spherical distributions allow more flexibility than Gaussian stimuli. They may serve, for example, to provide contrast normalization for each presented stimulus or to use stimulus distributions that drive the investigated neurons more efficiently than a Gaussian stimulus does (Ringach et al. 1997).

For the visual system, an interesting scenario might be the investigation of flashed images (Gollisch and Meister 2008b) or images that are presented in a saccade–fixation context (Segev et al. 2007). Other relevant scenarios may come from experiments where the temporal domain is intrinsically less relevant because of slower data acquisition. This may occur, for example, when analyzing data from calcium imaging, voltage-sensitive-dye imaging, or similar experiments. Here, spike-triggered analyses might be transformed into fluorescence-triggered analyses: Instead of selecting stimulus segments based on whether they elicited a spike or not, all stimulus segments are considered, but weighted by the elicited response strength, i.e. the fluorescence signal. This is analogous to working with trial-averaged firing rates or intracellularly measured synaptic currents (Demb 2008; Schwartz and Rieke 2011). As the temporal resolution of fluorescence imaging experiments is often not sufficient for analyzing temporal stimulus integration characteristics, it makes sense to present stimuli in a one-by-one fashion instead of a continuously updating time series.

Finally, even temporal stimulus attributes may be analyzed with non-Gaussian spherical stimulus distributions if a continuous stimulus update is not required. One such scenario occurs when individual stimulus segments are locked to external events such as a saccade (Geffen et al. 2007) or a contrast switch (Baccus and Meister 2002).

### Connection to Wiener series

STA and STC analyses are closely connected to the theory of Wiener series (Wiener 1958), which can be used to model the stimulus–response relation of a neuron by the first few terms of a functional expansion. For Gaussian white noise stimulation, this expansion can be systematically obtained by the Lee–Schetzen method, which derives the kernels of the Wiener series from various crosscorrelations between the stimulus and the response (Lee and Schetzen 1965), similar to the reverse correlation in STA and STC analysis. In fact, the first-order kernel of the Wiener series corresponds to the STA, and the second-order kernel of the series is obtained from the STC matrix.

For non-white Gaussian stimuli, this analogy still holds. The crosscorrelation method for obtaining the kernels of the Wiener series has been extended analogously to the derivation shown here in Section 4: apply a whitening transformation, obtain the Wiener kernels in the transformed space, and translate the results back to the original space, which in the case of Wiener series means to prepend the kernel operations with the whitening transformation (Lee and Schetzen 1965; Schetzen 1974).

Furthermore, efforts have been made to extend the method of Wiener series expansion or develop analogous functional expansion strategies for various types of non-Gaussian stimuli, such as spike-like inputs (Krausz 1975), noise stimuli with discrete input levels (Marmarelis 1977), superpositions of sinusoids (Victor and Knight 1979), and certain nonlinearly transformed Gaussian stimuli (Schetzen 1981). However, comparing these approaches to the treatment of non-Gaussian inputs in Section 3 highlights an important difference between the functional series models and the LN-model-based approach as in the present work. For the latter, the goal is generally to find the filters $\boldsymbol {k}_m$ that describe the system’s response according to Eq. (5), independently of the applied stimulus. This means that ideally the exact same relevant space should be obtained if the system is probed with different stimulus distributions. By contrast, the functional series models typically aim at minimizing the quadratic error between the predicted and the actual response for a given stimulus and a given order of the series expansion. The optimal kernels, which achieve this minimal error, may well depend on the applied stimulus, in particular for non-Gaussian stimuli where the constraints on the prior stimulus distribution might actually be exploited for response prediction. It is thus not surprising that analogies between Wiener series and STA and STC analysis for non-Gaussian stimuli appear less straightforward; yet, further exploration of the relation between these approaches for different stimulus distributions should prove a promising route for future investigations to arrive at a deeper understanding of the scope of these methods.

### A diversity of approaches in STC analysis

STC analysis has been formulated in several different versions, both in this work and in several previous studies. These different approaches are, of course, related to each other, though not always equivalent. In the following, we discuss some of their connections.

#### STC with and without subtracting the STA

Not surprisingly, the STA always lies in the relevant subspace, as shown in Section 3.2. This means that STC analysis can be performed with the actual covariance matrix of spike-triggered stimuli as well as with a variant of this matrix where the STA is not subtracted. The latter might be referred to as a matrix of second moments rather than an actual covariance, and it has also been termed “non-centered spike-triggered covariance matrix” (Cantrell et al. 2010). While both variants generally identify the correct relevant subspace, the obtained eigenvalue spectra and individual eigenvectors are typically different, as exemplified in Fig. 4. Comparing the spectra of STC analysis with and without subtracting the STA may thus serve as a simple consistency check and flag certain cases where one of the approaches fails to identify all relevant directions because the corresponding eigenvalue happens to coincide with the baseline of the irrelevant spectrum. As mentioned earlier, on the other hand, such a scenario is typically also picked up by explicitly including the STA when determining the relevant subspace, or more generally, by using an estimator that directly combines information from changes in stimulus mean and variance (Pillow and Simoncelli 2006).

#### *Relevant directions as eigenvectors of *Δ*C*

In several previous studies (Brenner et al. 2000; Agüera y Arcas and Fairhall 2003; Agüera y Arcas et al. 2003; Fairhall et al. 2006; Maravall et al. 2007), covariance analysis was based on diagonalizing the matrix Δ*C* = *C*_*s*_ − *C*_*p*_. For spherical stimulus distributions with unit variance of each stimulus component, *C*_*p*_ is the identity matrix. The eigenvectors of Δ*C* thus coincide with those of *C*_*s*_, and the eigenvalues are shifted downwards by one unit. The relevant and irrelevant directions of Δ*C*, hence, coincide with those of *C*_*s*_.

For a Gaussian elliptic stimulus distribution, Δ*C* still identifies the relevant subspace correctly (Bialek and de Ruyter van Steveninck 2005); the obtained relevant eigenvectors simply need to be premultiplied by $C_p^{-1}$ to correct for the correlations of the prior stimulus distribution. The eigenvalue spectrum and the individual eigenvectors now typically differ from those obtained with the procedures discussed in Section 4. Yet, the relevant spaces obtained with both methods coincide. Interestingly, we have seen above that relevant and irrelevant directions transform differently. The difference also appears when diagonalizing Δ*C*, since irrelevant directions must be premultiplied by *C*_*p*_, and not by $C_p^{-1}$. This ensures that relevant directions remain orthogonal to irrelevant directions.

The investigation of elliptic stimulus distributions through Δ*C* is only guaranteed to work with Gaussian stimulus distributions. As shown in Fig. 7, elliptic non-Gaussian stimulus distributions can lead to spurious eigenvalues deviating from the zero baseline level with this approach.

#### Relevant directions as stimulus directions of modified variance

A relevant eigenvector $\boldsymbol {w}$ fulfills the equation $C_p^{-1} C_s \boldsymbol {w} = \lambda \boldsymbol {w}$. With a little bit of algebra, it follows that the associated eigenvalue is $\lambda = \left(\boldsymbol {w}^T C_s \boldsymbol {w}\right)/\left(\boldsymbol {w}^T C_p \boldsymbol {w}\right)$. This expression represents a ratio of variances (Schwartz et al. 2006): the variance of the spike-eliciting stimuli to the variance of the prior stimuli, both measured along the direction of $\boldsymbol {w}$. Thus, searching for directions in stimulus space where this ratio is “unusual”, meaning that it differs from the baseline of variance ratios of irrelevant directions, serves as a way to identify relevant stimulus directions. This procedure is equivalent to the eigenvalue analysis of $C_p^{-1} C_s$.

Covariance analysis reveals changes in variances and is thus sensitive only to modifications up to the second moment of the probability distribution; higher order effects are not considered. Conversely, if for Gaussian stimulation the variance is altered in direction $\boldsymbol {k}_m$, then for sure this direction belongs to ${\cal K}$. Hence, the detectability by covariance analysis is a sufficient, but not a necessary condition, for a direction to be relevant in the sense of Eq. (5).

### Further characterization of the relevant space

As mentioned before, Eq. (5) defines the relevant space ${\cal K}$ unambiguously, but different sets of the individual filters $\boldsymbol {k}_m$ may be chosen without affecting the final spike probability. Even so, one may sometimes be interested in distinguishing between different directions inside ${\cal K}$. In some cases, additional structure within the relevant subspace may suggest a particular choice of the filters. As an example, cluster analysis of spike-triggered stimuli has been used to find filters within the relevant subspace that likely match actual physiological pathways (Fairhall et al. 2006; Geffen et al. 2007; Gollisch and Meister 2008a).

Alternatively, relevant directions may be distinguished based on the magnitude and sign of the change in variance along each direction. The latter has been used to classify relevant directions as either excitatory or suppressive, depending on whether the variance of spike-triggered stimuli is increased or decreased compared to the prior stimulus (Schwartz et al. 2002; Rust et al. 2004, 2005; Simoncelli et al. 2004; Schwartz et al. 2006). The classification into excitatory and suppressive stimulus directions through the magnitude of the eigenvalue makes sense only for relevant directions that are perpendicular to the STA. The STA itself, which usually functions as an excitatory stimulus direction, can be associated with an increase or decrease in variance, depending on the nonlinearity *φ*. Therefore, in those studies, the STA is typically projected out from each stimulus vector in order to then determine relevant directions orthogonal to the STA.

When the stimulus is not Gaussian, however, the size of an eigenvalue also reflects the effect of the interference between different stimulus directions imposed by the constraints of the prior stimulus distribution. For example, changing the nonlinearity along one relevant direction typically affects also the eigenvalues of other relevant directions. It is thus less straightforward to distinguish between excitatory and suppressive directions depending on the size of the eigenvalue. Yet, for most practical purposes, a distinction based on whether eigenvalues of relevant directions lie above or below the baseline level of irrelevant directions should provide a useful classification in terms of the excitatory or suppressive nature of relevant directions, given the constraints of the particular prior stimulus distribution.

Further characterization of the relevant space can come from observing degeneracies in the eigenvalue spectrum. These reflect fundamental properties of the firing probability, at least as long as the prior stimulus distribution is spherically symmetric. Even within the relevant space, eigenvalue degeneracies may be informative. A degeneracy in two or more relevant directions implies that the firing probability is endowed with additional symmetry properties: The variance is equally altered in several relevant directions. The eigenspaces associated with those symmetries are fundamental characteristics of the firing probability. Examples of such degeneracies in the relevant space have been found. Modeling studies have shown that resonator neurons are sometimes only selective to the stimulus frequency, but not to its phase (Mato and Samengo 2008). In such cases, covariance analysis detects a degenerate two-dimensional relevant eigenspace, generated by two periodic eigenvectors with a 90° phase-shift. Linear combinations of these two eigenvectors generate a periodic stimulus with arbitrary phase. Experimental studies of visual neurons in the fly (Bialek and de Ruyter van Steveninck 2005) and complex cells in mammalian visual cortex (Touryan et al. 2002; Rust et al. 2005) have reported similar degeneracies; the relevant space contained degenerate, two-dimensional eigenspaces, characterized by well-defined location, orientation, and frequency, with arbitrary phase.

## Conclusion

Here we have provided a geometric proof of consistency of spike-triggered covariance analysis. The geometric approach has led to an extension of the technique to arbitrary (non-Gaussian) elliptic stimulus distributions. For spherical distributions, irrelevant directions typically constitute a large degenerate eigenspace of the spike-triggered covariance matrix. Relevant directions are detected as the eigenvectors whose eigenvalues depart from the baseline degenerate level. In contrast to the Gaussian case, the value of irrelevant eigenvalues is not known a priori; it depends on the nonlinearity *φ*. For elliptic stimulus distributions, STC analysis can be appropriately modified to account for the correlations in the stimulus. This can be achieved by performing eigenvalue analysis on a matrix equal to the product of the inverse of the prior covariance matrix and the spike-triggered covariance matrix.

## Appendix A

Using group-theoretical arguments, here we prove that, for spherical stimulus distributions, the irrelevant subspace ${\cal K}_\perp$ is an eigenspace of *C*_*s*_. The prior distribution $P( \boldsymbol {s} )$ only depends on the length of $ \boldsymbol {s} $ and therefore remains invariant under any orthogonal transformation of the $ \boldsymbol {s} $-space, that is, any transformation that preserves the lengths of all vectors. Orthogonal transformations fulfill the condition *O* ^*T*^ = *O* ^− 1^. In addition, any transformation *O*_*i*_ acting only on the irrelevant subspace ${\cal K}_\perp$ also leaves the spike probability $\varphi(\boldsymbol {k}_1^T \  \boldsymbol {s} ,\ \dots,\ \boldsymbol {k}_M^T \  \boldsymbol {s} )$ invariant:

31 $$ \varphi\left(\boldsymbol {k}_1^T  \boldsymbol {s} , \dots, \boldsymbol {k}_M^T  \boldsymbol {s} \right) = \varphi\left(\boldsymbol {k}_1^T O_i  \boldsymbol {s} , \dots, \boldsymbol {k}_M^T O_i  \boldsymbol {s} \right). $$

There are many such orthogonal transformations *O*_*i*_. For example, all rotations whose rotation plane is contained in the irrelevant space and all reflections that invert stimulus directions within the irrelevant space leave *φ* invariant. These transformations, together with the ones obtained by combining them, constitute a group: the symmetry group of $P( \boldsymbol {s}  | {\rm spike})$. More formally, these transformations define a representation of this symmetry group. Since there is no proper subspace of ${\cal K}_\perp$ that remains invariant under the action of all the *O*_*i*_ of the group, the irrelevant space is an irreducible space of the representation.

The transformations *O*_*i*_ only operate in the irrelevant space and thus leave the relevant vectors invariant. In particular, the STA is left unchanged: Starting from the integral in Eq. (12) and introducing an orthogonal change of variables $ \boldsymbol {s}  = O_i  \boldsymbol {s} ^\prime$, we arrive at

32 $$ \langle  \boldsymbol {s}  \rangle = O_i \langle  \boldsymbol {s}  \rangle. $$

Using this same change of variables in the calculation of $\langle  \boldsymbol {s}   \boldsymbol {s} ^T \rangle$, we see that

33 $$ \left\langle  \boldsymbol {s}   \boldsymbol {s} ^T \right\rangle = O_i \left\langle  \boldsymbol {s}   \boldsymbol {s} ^T \right\rangle O_i^{-1}. $$

Therefore, the matrix *C*_*s*_ commutes with *O*_*i*_,

34 $$ C_s = \left\langle  \boldsymbol {s}   \boldsymbol {s} ^T \right\rangle - \langle  \boldsymbol {s}  \rangle \langle  \boldsymbol {s} \rangle^T= O_i \ C_s \ O_i^{-1}. $$

In group theory, Schur’s lemma states that if an operator commutes with all the matrices of an irreducible representation, then, inside the irreducible space of the representation, the operator is proportional to the unit matrix (Wiegner 1959; Tinkham 1964). Therefore, ${\cal K}_\perp$ must be an eigenspace of *C*_*s*_.

To illustrate the application of Schur’s lemma in this case, we note that if $\boldsymbol {v} \in {\cal K}_\perp$ is an eigenvector of *C*_*s*_ with eigenvalue *λ*, then Eq. (34) implies that $O_i \boldsymbol {v}$ is also an eigenvector of *C*_*s*_ with eigenvalue *λ*. By appropriately choosing *O*_*i*_, any vector $\boldsymbol {v}^{\prime} \in {\cal K}_\perp$ whose length is equal to $|\boldsymbol {v}|$ can be written as $\boldsymbol {v}^{\prime} = O_i \boldsymbol {v}$. Therefore, the whole of ${\cal K}_\perp$ is an eigenspace of *C*_*s*_. The same reasoning can be applied to the STC matrix when the spike-triggered average is not subtracted.

As a final remark, we point out that if $P( \boldsymbol {s}  | {\rm spike})$ also contains a symmetry group inside the relevant space and if the associated irreducible space has dimension larger than 1, degeneracies also appear in ${\cal K}$. Of course, $P( \boldsymbol {s}  | {\rm spike})$ might have no symmetry in the relevant subspace. But if, for example, $\varphi(\boldsymbol {k}_1^T \  \boldsymbol {s} ,\ \dots,\ \boldsymbol {k}_M^T \  \boldsymbol {s} )$ only depends on $(\boldsymbol {k}_1^T \boldsymbol {s} )^2 + (\boldsymbol {k}_2^T \boldsymbol {s} )^2 + \dots (\boldsymbol {k}_M^T \boldsymbol {s} )^2$, then when the prior stimulus distribution is spherical, also the relevant directions have degenerate eigenvalues. In Fig. 5, for example, the relevant eigenvalues of *C*_*s*_ are degenerate. This is not the case in Fig. 4, where the firing probability is not symmetric. In Fig. 6, instead, the firing probability is indeed symmetric, but the prior stimulus is not. Thus the degeneracy with respect to the relevant eigenvectors of $C_p^{-1}C_s$ is broken. In Fig. 7, the degeneracy is recovered, since the prior stimulus distribution is elliptic, but is (almost) spherical in ${\cal K}$.

## Appendix B

In Section 4, we extended STC analysis to general elliptic stimulus distributions. This extension was based on a transformation to a spherically symmetric stimulus distribution, for which the previously derived results were applicable. Here we provide the analogous extension for the STA.

When the spike probability *φ* contains a single relevant direction $\boldsymbol {k}_1$, the STA is proportional to $\boldsymbol {k}_1$ if the stimulus distribution is spherical (Chichilnisky 2001). If the stimulus distribution is elliptic, new variables $ \boldsymbol {s} ^{\prime}$ can be defined via Eq. (22), such that the prior stimulus distribution $P( \boldsymbol {s} ^{\prime})$ is spherical. In the transformed stimulus space, the STA

35 $$ \langle  \boldsymbol {s} ^\prime \rangle = \int {\rm d} \boldsymbol {s} ^\prime \ P( \boldsymbol {s} ^\prime | {\rm spike} ) \  \boldsymbol {s} ^\prime $$

is then proportional to the filter,

36 $$ \boldsymbol {k}_1^{\prime} \propto \langle  \boldsymbol {s} ^{\prime} \rangle . $$

In order to obtain the relation of STA and filter in the original space, we note that, because the integral in Eq. (35) can be transformed using $ \boldsymbol {s} ^{\prime} = D^{-1/2} O^T  \boldsymbol {s} $ and ${\rm d}  \boldsymbol {s} ^\prime P( \boldsymbol {s} ^\prime | {\rm spike}) = {\rm d} \boldsymbol {s}  P( \boldsymbol {s}  | {\rm spike})$, the transformation rule for the STA is the same as for individual stimuli, Eq. (22),

37 $$ \langle  \boldsymbol {s} ^\prime \rangle = D^{-1/2}\ O^T \ \langle  \boldsymbol {s}  \rangle . $$

Furthermore, the backward transformation for $\boldsymbol {k}_1^\prime$ is that of a relevant stimulus direction, Eq. (28),

38 $$ \boldsymbol {k}_1 = O \ D^{-1/2} \ \boldsymbol {k}_1^\prime , $$

so that the scalar products $\boldsymbol {k}_1^T  \boldsymbol {s} $ are preserved. Putting Eqs. (36)–(38) together, we obtain

39 $$ \boldsymbol {k}_1 \propto O \ D^{-1/2} \ D^{-1/2} \ O^T \ \langle  \boldsymbol {s}  \rangle = C_p^{-1} \ \langle  \boldsymbol {s}  \rangle . $$

We therefore arrive at the well-known recipe for estimating the single relevant direction by premultiplying the STA by the inverse of the prior covariance matrix (Theunissen et al. 2001; Paninski 2003; Schwartz et al. 2006). The derivation is valid for any elliptic stimulus distribution, not necessarily Gaussian. However, beyond elliptic stimulus distributions (e.g., natural stimulation), the premultiplication by $C_p^{-1}$ still does not generally suffice to obtain the relevant filter from the STA (Sharpee et al. 2004).
